# No-Harm Missing-Friction Correction for Potential-of-Mean-Force-Matched Coarse Graining

**DOI:** 10.1021/acsomega.6c07213

**Published:** 2026-08-19

**Authors:** Chunlin Zhao, Liwen Liu, Dongshi Zuo, Rigeng Wu, Liqian Zhang, Yongan Zhang, Yingchun Guo, Meian Li

**Affiliations:** † College of Computer and Information Engineering, 117454 Inner Mongolia Agricultural University, Hohhot 010018, China; ‡ Inner Mongolia Key Laboratory of Big Data Research and Application in Agriculture and Animal Husbandry, 117454 Inner Mongolia Agricultural University, Hohhot 010018, China

## Abstract

Potential-of-mean-force (PMF)-matched coarse graining (CG) can make a retained coordinate structurally correct but dynamically wrong. Eliminated bath degrees of freedom carry projected memory and friction that control response curves, relaxation times, kinetics, and transport. We ask whether the observable part of this missing friction can be recovered cheaply without harming generated dynamics. Residual-force fits and scalar rescalings alone cannot answer that question because they may expose the signal or improve one summary while worsening another. We distinguish the measured finite-window error between the high-resolution reference (labeled AA throughout) and CG dynamics from the component supported by residual-memory diagnostics, formulate that component as an observable projected-memory object, and approximate its consequence with a finite-history or surrogate candidate. The candidate is deployed only when held-out continuation rollouts improve both response-curve and relaxation-time errors. In two complete experiments, the fitted increment was added to the same detailed-balance PMF transition used as the uncorrected baseline. For C6 and C5 end-to-end distances, selected strength 0.10 retained PMF histogram L1 distances of 0.054 and 0.061, and the one-sided 5% lower bounds of both dynamical error reductions were positive. Separate precorrection diagnostics assigned acetone C–O to a richer state description and ethanol heavy-atom end-to-end to a finite memory kernel, so neither received the low-cost correction. The 13-coordinate breadth panel and diagnosis-informed tests provide breadth and selectivity evidence. Against a C6-calibrated scalar clock, the finite-history candidates improved both errors in 4/4 point-estimate comparisons versus 3/4 and gave 2.8 times the mean joint gain; experiment-specific uncertainty is reported separately. A distinct OpenFF 2.3.0/OpenMM surrogate showed the same point-estimate improvement on four molecules. The resulting reproducible, cost-bounded rule corrects only when structural and rollout evidence support correction.

## Graphical abstract

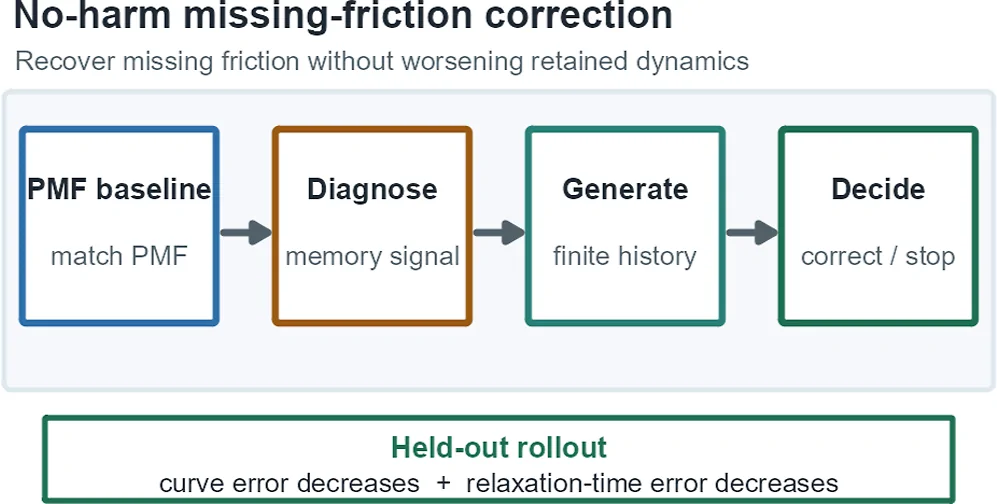

## 1 Introduction

Coarse-grained (coarse graining (CG)) molecular simulation is valuable because it replaces many atomistic degrees of freedom with a smaller set of retained coordinates. This economy makes relaxation, transport, and conformational response accessible on longer time and length scales.^1–4^ The same economy creates the specific failure addressed here. Bottom-up or potential-of-mean-force (PMF) matching can reproduce the equilibrium distribution or mean force along a retained coordinate,^3,5,6^ but the discarded atomistic variables still carry fast and orthogonal motions that shape friction, response curves, and relaxation times. A CG trajectory can therefore be structurally convincing while relaxing too quickly or distorting the time-domain observable it was built to predict. This loss of molecular roughness and dynamical resistance is a known source of artificially accelerated or mis-scaled CG motion.^7–11^ For kinetics, relaxation, and transport, this is not a cosmetic mismatch: the reduced model can be right in structure but wrong in the quantities that determine rates, mobilities, and response.

The central problem is therefore practical and testable: after PMF matching, the lost frictional content relevant to a declared coordinate must either be recovered at a small added cost or identified as outside a cheap correction’s boundary. This cannot be solved by simply fitting more residual force or by moving every coordinate to a full memory model because either choice can erase the cost advantage that motivated CG. The test must cover chemically distinct motions such as alkane chain distances, aromatic substituent motion, ketone internal motion, peptide-like local coordinates, and explicit boundary comparisons. It must also say when not to correct.

Projection theory identifies the missing object before any surrogate model is chosen. Eliminating atomistic variables does not leave only a mean force; it also leaves projected fluctuating forces and memory terms that enter the retained-coordinate dynamics.^12–15^ PMF matching keeps the equilibrium mean contribution but can omit the observable part of this bath memory. When residual-memory diagnostics support this mechanism, we call its observable consequence a missing-friction error. The AA–CG response difference alone is not a unique mechanistic diagnosis; it is the measured finite-window target for a declared coordinate, observable, and validation window. This distinction is what makes a low-cost solution possible: the correction need not reconstruct every unresolved force, only enough omitted bath response to improve the generated dynamics.

Existing dynamical CG corrections solve important parts of this problem, but they leave the decisive step open. Scalar time rescaling and roughness-based scaling can recover some average mobility or relaxation summaries.^7,8,10,16,17^ Dynamic force matching, dissipative parametrization, and generalized Langevin approaches add force, friction, or memory information.^18–22^ These methods establish that missing dynamics are real and can be represented. Representation, however, is not deployment. A residual-force fit can reveal a predictable signal while the generated trajectory still worsens relaxation. A single time-scale factor can help one summary while damaging curve shape or transfer to another coordinate. Full memory-kernel models can be more expressive, but they also move the calculation into a more expensive modeling class. The missing element is a same-data rule that decides when a low-cost correction is worth using, when it should be rejected, and when the coordinate should be escalated. Recent probabilistic forecasting, internal-friction, and multidimensional-GLE studies further extend these richer representation classes.^23–25^

We address this gap by making deployment rather than residual fitting the object of the method. For each declared coordinate and validation window, the theoretical target is the observable projection of the residual fast-bath memory. The practical operator is a finite-history or a surrogate correction. It is allowed to deploy only after withheld reference vs reduced process rollouts improve both the response curve error and the relaxation time error. The decision rule therefore has four outcomes: correct, abstain, reject the candidate, or escalate to a richer memory model. Rejection and abstention are part of the method because they prevent a cheap correction from being used outside the memory class and observable for which it improves the final dynamics.

The contribution has three linked parts. First, we define the post-PMF error as an observable projected memory object rather than a generic residual force score. Second, we turn that object into a no-harm deployment rule: a fixed finite-history or surrogate correction is accepted only if withheld continuation trajectories improve both response-curve and relaxation-time errors. In the complete molecular experiments, the same PMF transition is used to define the uncorrected error, receive the correction, and provide the comparator, and the corrected trajectory must retain the fitted PMF distribution. Third, we evaluate the decision against a C6-calibrated scalar transfer baseline at an explicit added cost. This separates the work from residual signal detection without generated trajectory evidence, scalar time rescaling without memory diagnosis, and richer memory fitting without an explicit cost boundary. The test concerns the chemically coordinated response produced by the CG model and not whether a feature model can fit an isolated residual signal.

Accordingly, Section 2 defines the abstract objects and decision, Section 3 instantiates them for molecular coordinates, and Section 4 reports correction or early stopping before the postdecision scalar cost comparison.

The evidence is tiered rather than pooled. C6 and C5 are complete same-PMF correction experiments; acetone C–O and ethanol heavy-atom end-to-end test applicability before correction; the 13-coordinate and diagnosis-matched tests provide supporting breadth and selectivity. The OpenFF 2.3.0/OpenMM panel tests the same two-observable decision criterion through a distinct stochastic two-lag surrogate; it does not test numerical transfer of the additive kinematic/QVA implementation. Figure 1 summarizes both the method-to-evidence chain and the distinct experiment identities behind repeated C6 and C5 coordinate labels.

**Figure 1:**
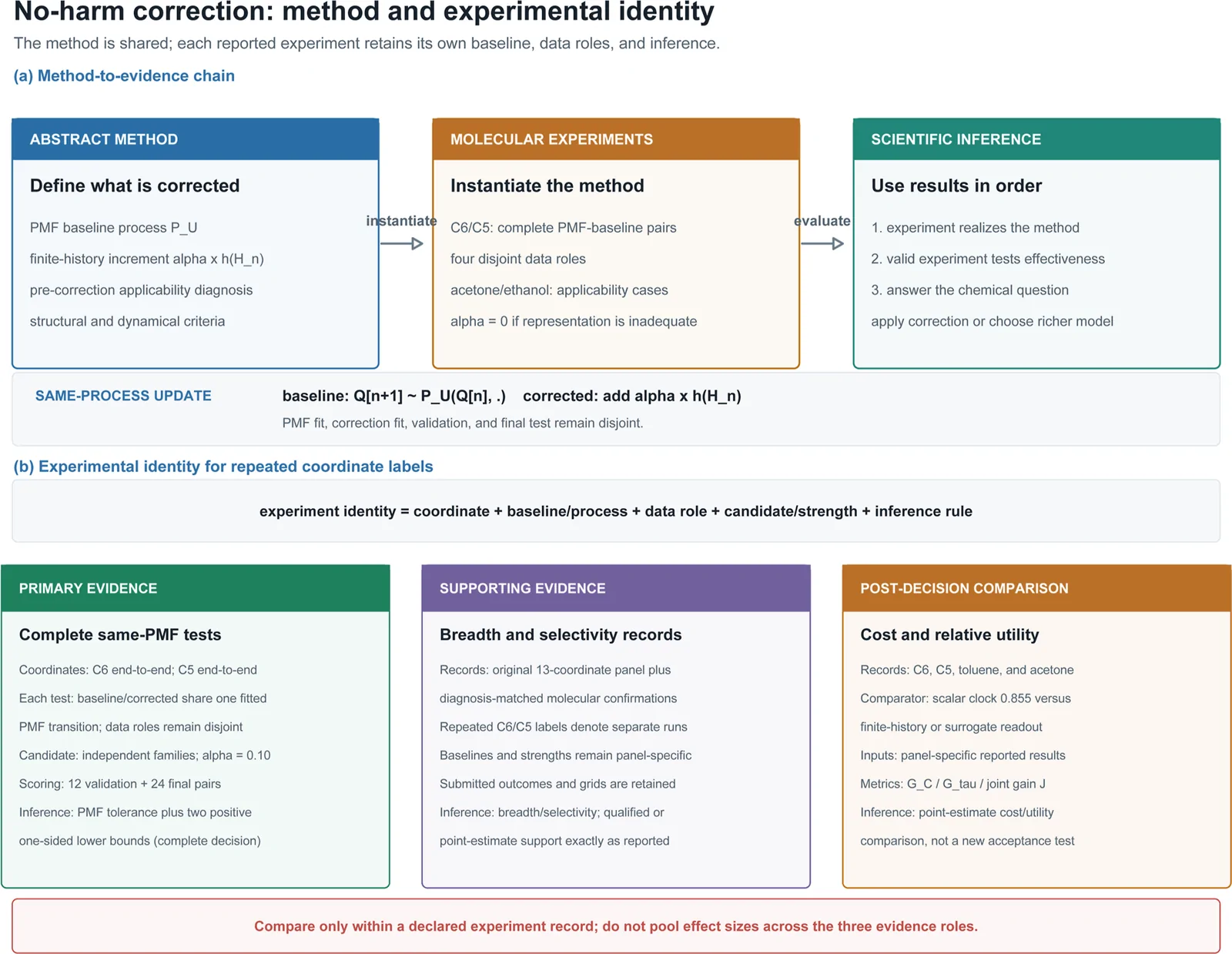
Method and experimental identity of the finite-history no-harm correction. (a) The abstract method defines a PMF baseline process, a finite-history increment, applicability diagnostics, and structural and dynamical requirements; the molecular calculations instantiate those objects before scientific inference. (b) Repeated coordinate names do not denote one pooled experiment. Within each complete C6/C5 test, baseline and corrected replicas share one fitted PMF transition and use disjoint data roles, independently selected candidates at α = 0.10, and paired lower-bound decisions. The 13-coordinate breadth and diagnosis-matched records retain their panel-specific baselines, strengths, and evidence qualifications. The postdecision cost table reuses its declared point-estimate readouts for relative utility; it is not a new acceptance test. Effect sizes are compared only within their declared experiment records.

## 2 Missing-Friction Error Object and Deployment Rule

The three contributions stated in the Introduction are implemented through four operational stages. Stage 1 qualifies the panel-specific PMF prerequisite and declares the measured post-PMF error . $C_{T}$ stage 2 assigns a diagnosed memory class and formulates the observable projected-memory object $O_{Q}$ . Stage 3 approximates its observable consequence by a declared finite-history map $h_{H}$ or surrogate and fixes at most one candidate using training and validation data. Stage 4 applies the held-out rule to return the action *â*. Thus, the measured error, diagnosed target, theoretical object, numerical candidate, and final action are related but not interchangeable. The exact finite-history fit and recursive rollout are given below.

Let *Q*_t_ denote the CG coordinate and let *C*_Q_(*t*) denote the time-correlation observable used for validation. For the declared window [0, *T*], the normalized weight is *w*(*t*) = 1/*T*. A PMF-only reduced model can match the equilibrium distribution of *Q* while producing a different finite-window observable

1 $$C_{T}\left(Q\right)=\int_{0}^{T}w\left(t\right){\left|C_{Q}^{AA}\left(t\right)-C_{Q}^{CG}\left(t\right)\right|}^{2}dt$$

We define the missing-friction target as the projected-memory part of this finite-window observable error when the residual-memory diagnostics support that attribution. Because the measured AA–CG discrepancy can also contain baseline, discretization, or other reduction error, it is not by itself a unique mechanistic diagnosis. We therefore fix the hypothesized source before training and target only the omitted bath response that changes the declared time-domain observable. PMF agreement alone is not a dynamical criterion: the observable, window, and post-PMF rollout error in eq 1 must be declared before correction.

### 2.1 Observable Projected-Memory Object

At retained-coordinate value *q*, let *S*_q_ denote the integrated conditional transport of the eliminated bath and *S*_q_^0^ the part represented by the declared PMF-only process. If *A*(*q*) maps locally centered bath variation to unresolved force along *Q*, projecting the residual transport onto the declared response gives

2 $$O_{Q}=\Pi_{obs}\left[\beta A\left(q\right)\Delta S_{q}A{\left(q\right)}^{Τ}\right],,\Delta S_{q}=S_{q}-S_{q}^{0},,\beta ={\left(k_{B}T\right)}^{-1}$$

where Π_obs_ retains only the component that changes *C*_Q_(*t*) on [0, *T*]. Thus, $O_{Q}$ is an operator-level dissipative response, not a one-step coordinate increment. Conditional on the residual-memory diagnosis, the fitted finite-history increment is an empirical surrogate for its observable consequence; the calculation does not estimate *S*_q_, *S*_q_^0^, Π_obs_, or a unique microscopic decomposition. Supporting Information Section S2 gives the source dynamics, distance-coordinate map, conditional bath correlation, and Poisson-corrector representation. The construction follows Mori–Zwanzig memory, linear-response friction, and homogenization descriptions of eliminated variables.^12,13,15,26^

### 2.2 Numerical Realizations

The complete C6/C5 implementation below uses one detailed-balance PMF transition to define the target, generate the uncorrected comparator, and receive the finite-history increment. The 13-coordinate breadth analysis instead uses a zero-residual discrete comparator after separate PMF-transfer qualification; its regression, target, clipping, and recursive rollout are reported in Supporting Information, Section S4. It therefore establishes breadth and selection behavior, not corrected process PMF preservation. The OpenFF/OpenMM calculation uses a separate stochastic two-lag surrogate described in Section 3. It tests the shared two-observable decision criterion through a different surrogate and not numerical transfer of the additive implementation; the two realizations are not pooled as equivalent evidence.

### 2.3 Concrete PMF-Baseline Correction Process

Let *P*_U_(*i*,*j*) be the finite-volume transition fitted from the PMF-training block on bin centers *c*_i_, with invariant mass π_i_ and π_i_ *P*_U_(*i*,*j*) = π_*j*_ *P*_U_(*j*,*i*). Its conditional mean increment is

3 $${\mathrm{\Delta ̅}}_{U}\left(i\right)=\sum_{j}P_{U}\left(i,j\right)\left(c_{j}-c_{i}\right)$$

For the complete C6 and C5 experiments, the finite-history target is

4 $$y_{n+1}^{sp}=\left(Q_{n+1}^{AA}-Q_{n}^{AA}\right)-{\mathrm{\Delta ̅}}_{U}\left(I_{n}\right)$$

where *I*_*n*_ is the PMF-grid state nearest *Q*_*n*_. After fitting the predeclared feature vector $\phi_{H}$ on the correction-training block

5 $$\mathrm{\theta ̂}=\arg\min_{\theta }\sum_{n\in train}{\left[y_{n+1}^{sp}-\theta_{H}^{T\phi }\left(H_{n}\right)\right]}^{2}+\lambda ∥\theta {∥}_{2}^{2},,{ĥ}_{H}={\mathrm{\theta ̂}}_{H}^{T\phi }$$

with feature scaling, λ, and clipping fixed from training data. One common uniform draw then maps *I*_*n*_ to *J*_*n*+1_ ≈ *P*_U_(*I*_*n*_, ·), and the paired trajectories use

6 $$Q_{n+1}^{\left(\alpha \right)}=C_{U}\left[Q_{n}^{\left(\alpha \right)}+c_{J_{n+1}}-c_{I_{n}}+\alpha {ĥ}_{H}\left(H_{n}^{\left(\alpha \right)}\right)\right]$$

The baseline is the same equation with α = 0. Paired baseline and corrected replicas share the PMF kernel, invariant initial-state law, reflecting support, burn-in, and random numbers; only the fitted history increment differs.

For pooled generated histogram masses ${\mathrm{p̂}}_{i}$ , define

7 $$D_{PMF}=\sum_{i}\left|{\mathrm{p̂}}_{i}-\pi_{i}\right|=2D_{TV}\left(\mathrm{p̂},\pi \right)$$

A candidate is eligible only if *D*_PMF_ ≤ 0.10 for both baseline and corrected replicas and the one-sided 5% paired-bootstrap lower bounds of both *E*_C_^(0)^ – *E*_C_^(α)^ and *E*_τ_^(0)^ – *E*_τ_^(α)^ are positive. The structural threshold is a predeclared operational resolution tolerance on the fixed 160-bin partition, not a universal physical constant. It is equivalent to total-variation distance at most 0.05; consequently, for any [0, 1]-valued observable represented on that same partition, the expectation discrepancy under the generated and fitted PMF distributions is at most 0.05. Feature family and strength are fixed on validation replicas before the final replicas are generated.

### 2.4 Applicability and Theoretical Boundary

A memory class is the diagnostic assignment of the unresolved response; the feature family H is the numerical span used to construct a candidate. A low-cost correction is considered only when the PMF-only process represents the coordinate distribution, the residual contribution has finite memory on the declared window, and the sign, tail, time scale, and state diagnostics assign it to a class representable in H . The class was fixed before scoring. State switching, long tails, unstable signs, or hidden variables instead require a longer kernel, state conditioning, or additional variables.

Let $P_{H}O_{Q}$ be the best representation of the projected operator inside the declared feature span. The remainder $O_{Q}-P_{H}O_{Q}$ is the part that still requires a longer kernel, state conditioning, or hidden variables. The sufficient-margin expression is

8 $$m_{T}\left(h\right)=C_{T}\left(0\right)-C_{T}\left(h\right)>L_{T}∥O_{Q}-P_{H}O_{Q}∥+\varepsilon_{T}$$

with the same sign requirement imposed on the relaxation-time metric. Here *L*_T_ bounds the change in the declared finite-window response error per unit of operator-approximation error under the fixed baseline and window, and ϵ_T_ collects sampling and rollout uncertainty. Neither quantity is estimated as a numerical theorem here. The operational counterpart is to require positive held-out margins for both declared dynamical errors after the candidate class and strength have been fixed. Otherwise the process remains unmodified or is assigned to a richer model class.

### 2.5 Observable Acceptance and Four Actions

For δ*Q*_t_ = *Q*_t_ – ⟨*Q*⟩, define

9 $$C\left(t\right)=\frac{\left\langle \delta Q_{t}\delta Q_{0}\right\rangle }{\left\langle \delta Q_{0}^{2}\right\rangle }$$

The normalized curve error and relative relaxation-time error are

10 $$E_{C}=\frac{{\left[\int_{0}^{T}w\left(t\right){\left(C_{AA}\left(t\right)-C_{CG}\left(t\right)\right)}^{2}dt\right]}^{1/2}}{{\left[\int_{0}^{T}w\left(t\right)C_{AA}{\left(t\right)}^{2}dt\right]}^{1/2}}$$

11 $$E_{\tau }=\frac{\left|\tau_{CG}-\tau_{AA}\right|}{\left|\tau_{AA}\right|}$$

where τ is the trapezoidal integral over the first positive correlation lobe. For either error, *G* = 1 – *E*_candidate_/*E*_baseline_. We distinguish two evidence levels throughout. Uncertainty-qualified no-harm acceptance requires positive one-sided lower bounds for both error reductions under the stated sampling procedure. A point-estimate comparison records only that both observed errors decrease; it is not an uncertainty-qualified deployment result. Training constructs the candidate, validation selects at most one nonzero strength, and held-out scoring determines the final action. Let *B* denote PMF transfer, *M* the declared memory-class diagnostic, *S* selection of a nonzero candidate, *U* a predeclared validation-stage hard-safety veto (including an unstable response island), and *A* held-out empirical acceptance. The rule is

12 $$â=\{\begin{array}{ll} correct & B\cap M\cap S\cap U^{c}\cap A\\ abstain & B^{c}\text{or}\left(B\cap M\cap S^{c}\cap U^{c}\right)\\ reject & B\cap M\cap \left[U\text{or}\left(S\cap A^{c}\right)\right]\\ escalate & B\cap M^{c} \end{array}$$

Thus, a validation-stage hard-safety failure is a rejection even when the deployed strength remains zero; absence of a safe nonzero candidate without such a failure is abstention. Only correct returns a nonzero process. The no-harm claim is therefore conditional on the declared coordinate, window, candidate class, protocol, and held-out continuation.

## 3 Computational Methods and Molecular Validation Protocol

This section instantiates the abstract method in molecular calculations. The two complete experiments use C6 and C5 end-to-end coordinates: each fits one PMF process, defines the finite-history target relative to that process, and compares corrected and uncorrected continuations generated from the same PMF transition. Acetone C–O and ethanol heavy-atom end-to-end test applicability before correction. The 13-coordinate breadth panel, diagnosis-matched tests, the 45-coordinate inventory, and the OpenFF/OpenMM arm provide supporting breadth, boundary, and cross-system comparison evidence. These roles are reported separately. As Figure 1b makes explicit, a coordinate label alone does not identify an experiment: the baseline process, data role, candidate/strength, and inference rule together define the record.

### 3.1 Molecular Systems, Coordinates, and Baseline Qualification

Each reduced representation is one declared scalar coordinate *Q* = ξ­(*r*). The breadth panel contains 12 fixed distance coordinates from united-atom linear alkanes and one exploratory cyclohexane comparison; C5 and C6 denote five- and six-site linear alkanes. The higher-resolution references were generated with the stated molecular models using OpenMM Langevin-middle dynamics. Complete force-field parameters, coordinate pairs, trajectory lengths, output strides, seeds, and the separately parametrized cyclohexane comparison are given in Supporting Information Sections S2–S3 and the machine-readable registry.

For each coordinate, the PMF was estimated on a fixed 160-bin training support. A nearest-neighbor finite-volume Smoluchowski generator satisfies local detailed balance; its 0.01 ps transition must be row stochastic, preserve the discrete PMF distribution, and give a pooled fixed-grid histogram L1 distance no greater than 0.10 from the higher-resolution reference. The coordinate friction is fixed as γ_*Q*_ = μγ_AA_, rather than fitted to the relaxation error. Exact rates, boundary conditions, and qualification details are reported in Supporting Information Section S4.

The validation cost counts pretest fitting and selection frames, scalar scans, and held-out generated frames after a PMF-only process. Local postprocessing cost measures regeneration of the released comparison table and excludes production molecular dynamics. The method is therefore an inspection and correction layer for a declared reduced process and not a new force-field calibration procedure.

### 3.2 Diagnostics and Candidate Families

Residual force correlations and projected-memory checks diagnose an admissible memory class but do not authorize deployment. Target-informed upper bounds and candidate scans have the same diagnostic roles. The 45-coordinate inventory covers linear and cyclic hydrocarbons, alcohols, ketones, aromatics, and peptidic systems. It assigns each coordinate to one of four treatments: local short memory, a finite kernel, state conditioning, or richer hidden variables. The assignment preceded held-out scoring. The AA-target upper bound is nondeployable because it inserts the contemporaneous held-out target during recursive propagation.

The 13-coordinate breadth panel tests zero correction, three trainable four-lag families, and the nondeployable upper bound. Diagnosis-matched supporting tests use kinematic-plus-signed or coordinate–velocity–acceleration (QVA)-plus-signed finite-history features when predeclared by the diagnostic record. The former combines velocity lags with signed short-memory convolutions; the latter adds coordinate, acceleration, and mean conditional force. Exact feature vectors, memory horizon, regularization, clipping, candidate grids, selector thresholds, per-coordinate choices, and script entry points are given in Supporting Information Section S4. All choices precede held-out generated-trajectory scoring; scalar rescaling and cost comparisons are evaluated only after the correction decision.

### 3.3 Complete PMF-Baseline Correction Experiments

The complete experiments used C6 end-to-end with the kinematic-plus-signed family and C5 end-to-end with the QVA-plus-signed family. Each trajectory is divided into four nonoverlapping blocks for PMF fitting, correction fitting, strength selection, and untouched final scoring. Signed-memory construction, force conditioning, feature scaling, ridge coefficients, and clips use only the first two blocks; the exact index ranges are listed in Supporting Information, Section S4.

For each candidate strength, 12 paired validation replicas use common random numbers. The passing strength that maximizes the smaller of the two dynamical lower bounds is fixed. Final scoring uses 24 newly seeded paired replicas, a 20 ps burn-in, and 5000 paired-bootstrap draws. Baseline and corrected members of each pair share the detailed-balance PMF transition, invariant initial-state law, reflecting support, and uniform draws. Acceptance requires *D*_PMF_ ≤ 0.10 for both generated pools and positive one-sided lower bounds for both dynamical error reductions. The bootstrap quantifies generated-process variability against a fixed final reference block; it is not an independent molecular-ensemble certificate.

### 3.4 Precorrection Applicability Experiments

For acetone C–O and ethanol heavy-atom end-to-end, the projected residual force is $F_{⊥}=F_{Q}-E\left[F_{Q}|Q\right]$ . The force/coordinate time-scale ratio $\epsilon_{sep}=\tau_{F}^{+}/\left|\tau_{Q}\right|$ is compared with the fixed short-memory and finite-kernel limits 0.35 and 1.0. State dependence is measured by ϵ_state_, the interquartile range divided by the median of τ_F_^+^ across five equal-population *Q* bins, with fixed limit 1.0. These dimensionless cutoffs were predeclared for the breadth analysis and were not fitted to correction outcomes. The 0.35 cutoff requires the positive residual-force correlation time to remain within roughly one-third of the coordinate correlation time; 1.0 marks the loss of that time-scale separation, while ϵ_state_ = 1 means that the across-bin interquartile range equals the median memory time. They are operational limits for the tested low-cost classes rather than universal physical constants. These diagnostics are evaluated before any nonzero candidate is fitted. A coordinate outside the declared low-cost class is left at α = 0 and assigned to the required richer model; thresholds and calculations are detailed in Supporting Information Section S4.

### 3.5 OpenFF/OpenMM Finite-History Surrogate Arm

Acetone, toluene, ibuprofen, and cholesterol were parametrized with OpenFF 2.3.0 and sampled in gas phase using the CUDA platform in OpenMM. For standardized coordinate $z_{n}=\left(Q_{n}-{\mathrm{Q̅}}_{tr}\right)/s_{Q,tr}$ , the training half of each 20,000-frame trajectory fits

13 $$z_{n+1}=a_{1}z_{n}+a_{2}z_{n-1}+b+\sigma \varepsilon_{n+1},,\varepsilon_{n+1}∼N\left(0,1\right)$$

by ridge regression with 10^–6^ *I*; σ is the training residual standard deviation. The fitted process is initialized from the first two withheld values and recursively generates the 10000-frame comparison. A PMF-only process and its training-fitted scalar time rescaling are generated separately. This arm uses no kinematic/QVA feature vector or α grid. Its two-observable no-harm rule requires both curve NRMSE and relaxation-time relative error to be no worse than the PMF-only process. It tests an independently fitted stochastic AR(2) surrogate, not solvent, force-field-family, or long-time scale transfer.

### 3.6 Algorithm 1: PMF-Referenced Finite-History Correction

For one declared molecular coordinate, the complete correction and no-harm sequence is

1 Declare the molecular model, retained coordinate, fitted PMF process, response window, finite-history family H , candidate strengths, disjoint data blocks, and structural and dynamical tolerances were declared.
2 Fit the PMF transition on training data and the correction target relative to its conditional mean increment is defined by eq 4.
3 Diagnose residual-memory applicability before fitting a nonzero correction. Continue only when the declared low-cost family matches the diagnosis; otherwise leave α = 0 and use a finite-kernel, state-conditioned, or hidden-variable model.
4 Fit only the declared feature projections. On validation data, generate paired uncorrected and corrected candidates from the same PMF process by eq 6 and freeze at most one nonzero strength that preserves the PMF and improves both dynamical errors.
5 On the untouched final block, compare newly seeded paired replicas. Apply the correction only when the corrected PMF remains within tolerance and the one-sided lower bounds of both dynamical error reductions are positive; otherwise, leave the PMF process unmodified.

The zero-residual implementation used in the 13-coordinate breadth analysis is defined separately in Supporting Information, Section S4. Representative generated trajectory outcomes from this breadth analysis are shown in Figure 2.

**Figure 2:**
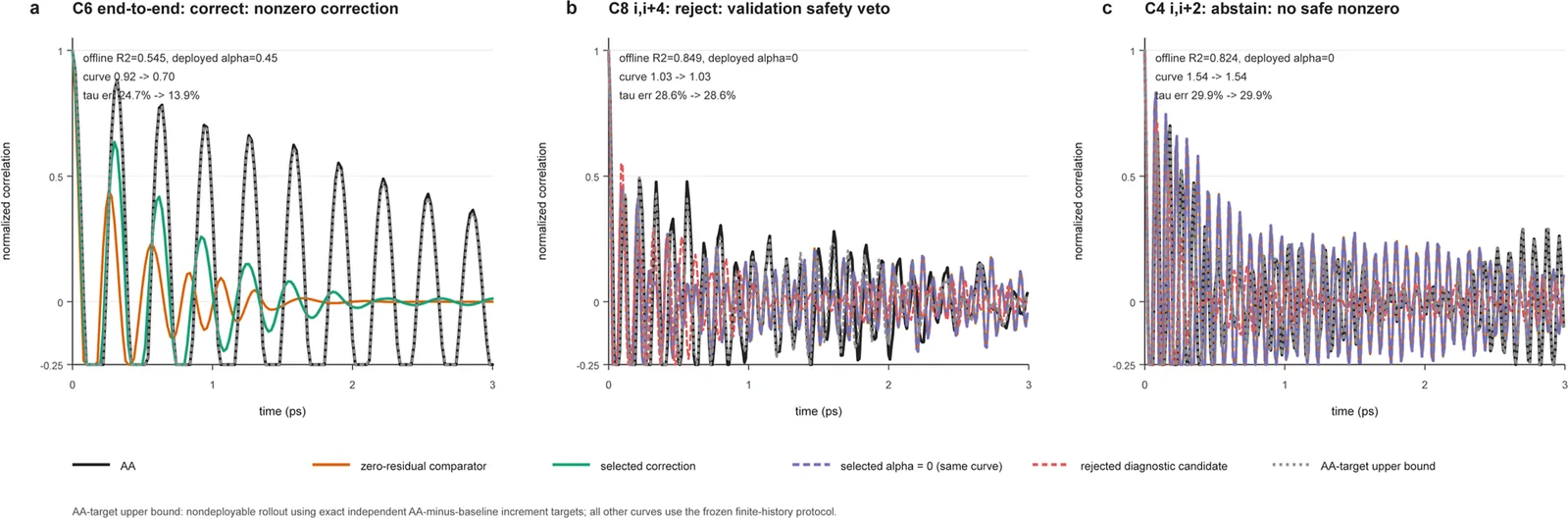
Supporting generated-trajectory results from the 13-coordinate breadth analysis. The C6 end-to-end candidate improves the time-correlation response relative to that panel’s zero-residual discrete comparator, whereas the other examples show why offline residual signal or target-informed AA-target upper-bound recoverability alone does not justify correction.

## 4 Results

Tables 1–9 report every numerical result used in the Discussion, grouped by experiment rather than by the order in which the method is executed. This keeps each comparator, candidate, result, and scientific interpretation together. Tables 1 and 2 are the complete same-PMF-process tests; later C6 and C5 rows report breadth-panel or diagnosis-informed calculations with their panel-specific comparators and strengths, so their effect sizes are not pooled.

**Table 1:** Complete C6 End-to-End PMF-Baseline Correction Experiment^a^

test	numerical result	interpretation
concrete implementation	kinematic-plus-signed candidate; α = 0.10; 12 validation and 24 final paired replicas	candidate fixed on validation; independent final comparison
PMF structure	*D*_PMF_ = 0.03456/0.05401, uncorrected/corrected	both below the predeclared 0.10 tolerance
correlation curve	*E*_C_: 0.97292 → 0.96805; *B*_C_ = 0.004446	positive lower bound
relaxation time	*E*_τ_: 0.77819 → 0.74176; *B*_τ_ = 0.033970	positive lower bound
final decision	PMF and both dynamical requirements pass	accepted no-harm correction

a The uncorrected and corrected replicas use one shared PMF transition component; *B*_C_ and *B*_τ_ are the one-sided 5% paired-bootstrap lower bounds of the absolute reductions in curve and relaxation-time error.

**Table 2:** Complete C5 End-to-End PMF-Baseline Correction Experiment^a^

test	numerical result	interpretation
concrete implementation	QVA-plus-signed candidate; α = 0.10; 12 validation and 24 final paired replicas	candidate fixed on validation; independent final comparison
PMF structure	*D*_PMF_ = 0.01204/0.06071, uncorrected/corrected	both below the predeclared 0.10 tolerance
correlation curve	*E*_C_: 0.95092 → 0.94896; *B*_C_ = 0.001739	positive lower bound
Relaxation time	*E*_τ_: 0.26908 → 0.23450; *B*_τ_ = 0.025972	positive lower bound
final decision	PMF and both dynamical requirements pass	accepted no-harm correction

a The uncorrected and corrected replicas use one shared PMF transition component; BC and Bτ are defined as in Table 1.

### 4.1 Complete PMF-Baseline Correction Experiments

Both complete experiments embedded the correction in the same qualified PMF transition used by the uncorrected process. The shared PMF transition component had a detailed-balance error below 1.8 × 10^–17^. Validation selected α = 0.10 for C6 and C5, and both final comparisons passed the PMF and two dynamical requirements in Tables 1 and 2. The structural test affected selection: at α = 0.25, both candidates still had positive dynamical lower bounds, but corrected PMF L1 distances increased to 0.10370 and 0.25342, so they were excluded.

### 4.2 Precorrection Applicability Results

The two applicability experiments stopped before correction for different physical reasons (Tables 3 and 4). Acetone C–O exceeded both separation limits and the state-variation limit, whereas ethanol heavy-atom end-to-end lay in the finite-kernel range. Thus, both retained α = 0, but the former requires a richer state or hidden-variable description and the latter a finite-memory kernel. Across the separate 45-coordinate inventory, 42 coordinates were assigned to an explicit diagnostic class (Table 5); this result measures diagnostic coverage rather than correction success.

**Table 3:** Acetone C–O Pre-Correction Applicability Experiment^a^

test	numerical result	interpretation
residual-memory diagnosis	ϵ_sep_ = 2.773; ϵ_state_ = 5.367	both declared limits are exceeded
method output	α = 0; no correction is fitted or generated	precorrection stop
scientific conclusion	the declared low-cost family is inapplicable	a richer state or hidden-variable description is required

a The declared short-memory limits are ϵ_sep_ ≤ 0.35 and ϵ_state_ ≤ 1.0.

**Table 4:** Ethanol Heavy-Atom End-to-End Pre-Correction Applicability Experiment^a^

test	numerical result	interpretation
residual-memory diagnosis	ϵ_sep_ = 0.890; ϵ_state_ = 0.230	finite-kernel range, outside the declared short-memory range
method output	α = 0; no correction is fitted or generated	precorrection stop
scientific conclusion	a local short-memory update is insufficient	a finite-memory kernel is required

a The finite-kernel separation limit is ϵ_sep_ ≤ 1.0.

**Table 5:** Forty-Five-Coordinate Applicability Inventory^a^

diagnostic class	coordinates	result
local short-memory	33	eligible for the declared low-cost family
finite-kernel memory	8	requires an explicit finite-memory kernel
state-conditioned	1	requires state-dependent treatment
abstain or richer representation	3	no correction attempted in the declared family
inventory total	45	42 assigned to an explicit diagnostic class

a This experiment tests diagnostic coverage and does not count as evidence of a successful correction.

### 4.3 Breadth-Panel Selectivity

All 13 coordinates passed PMF qualification, and their nondeployable AA-target upper bounds indicated substantial target-informed recoverability. Held-out-generated trajectories nevertheless returned only one nonzero correction, four zero-strength abstentions, and eight rejections (Table 6). C8 *i*,*i* + 4 illustrates why offline fit is insufficient: its *R* ^2^ exceeded that of C6, but both dynamical gains were negative. C4 *i*,*i* + 2 and cyclohexane showed curve-only improvements with harmful relaxation responses. The full machine-readable rows are indexed in Supporting Information Section S5.

**Table 6:** Thirteen-Coordinate Breadth and Selectivity Experiment^a^

coordinate	*R* ^2^	*U*_C_ (%)	α^★^	*G*_C_ (%)	*G*_τ_ (%)	decision/boundary
C6 end-to-end	0.545	98.07	0.45	20.87	51.45	correct; only nonzero result
C8 (*i*, *i* + 4)	0.849	71.39	0.45	–11.32	–13.20	reject; unstable response island
C4 (*i*, *i* + 2)	0.824	100.00	0.50	22.11	–1.15 × 10^3^	abstain at zero; harmful nonzero rejected
C5 (*i*, *i* + 2)	0.783	84.04	0.02	–9.49	–363.74	reject; unstable response island
C5 end-to-end	0.773	100.00	0.40	1.06	–10.59	reject; curve-only improvement
C8 end-to-end	0.713	78.71	0.45	8.30	–71.55	abstain at zero; relaxation harmed
C8 (*i*, *i* + 2)	0.672	92.23	0.45	26.19	–806.57	reject; unstable relaxation
C4 end-to-end	0.629	100.00	0.50	–1.32	–2.53 × 10^5^	reject; unstable response island
cyclohexane opposite-carbon	0.594	89.72	0.50	36.33	–5.59 × 10^3^	reject; curve-only failure
C6 (*i*, *i* + 4)	0.324	80.61	0.20	–24.59	–1.79 × 10^3^	reject; both criteria fail
C7 end-to-end	0.253	97.62	0.45	–3.47	–12.87	abstain at zero
C7 (*i*, *i* + 4)	0.272	99.64	0.45	–1.59	–477.19	reject; unstable response island
C10 end-to-end	0.247	98.86	0.50	–13.23	–39.94	abstain at zero

a *R* ^2^ is the offline dynamic score, *U*_C_ is the nondeployable AA-target upper-bound curve gain, α^★^ is the best tested nonzero strength, and *G*_C_/*G*_τ_ are the minimum late-window curve/relaxation-time gains. Large negative relative gains indicate unstable response or a small reference error denominator and are treated as failures, not stable effect sizes.

### 4.4 Targeted Confirmations

All four diagnosis-informed candidates improved both point-estimate errors (Table 7). Paired moving-block sensitivity retained positive one-sided 95% lower margins for both observables only for C5 and toluene. Alanine’s relaxation-time margin and both acetone margins crossed zero, so those two remain point-estimate confirmations rather than uncertainty-qualified results.

**Table 7:** Diagnosis-Informed Molecular Confirmations^a^

coordinate	candidate	errors, base → corrected	*G*_C_ (%)	*G*_τ_ (%)	sensitivity/decision
C5 end-to-end	local short-memory; QVA-plus-signed; α = 0.10	*E*_C_: 1.340 → 0.885; *E*_τ_: 19.72 → 18.93%	33.97	4.01	*L*_C_ = 0.0731; *L*_τ_ = 0.00213; *p*_+_ = 0.9580; qualified
toluene ring-methyl	state-sensitive; kinematic-plus-signed; α = 0.50	*E*_C_: 1.768 → 0.857; *E*_τ_: 78.54 → 77.03%	51.53	1.93	*L*_C_ = 0.255; *L*_τ_ = 0.0109; *p*_+_ = 0.9996; qualified
alanine cap distance	local short-memory; QVA-plus-signed; α = 1.00	point-estimate comparison	12.91	10.62	*L*_C_ = 0.0445; *L*_τ_ = – 0.00516; *p*_+_ = 0.6966; point estimate
acetone methyl–methyl	state-enriched QVAsurrogate; α = 0.50	*E*_C_: 3.298 → 2.848; *E*_τ_: 5.97 → 1.58%	13.62	73.49	*L*_C_ = – 0.448; *L*_τ_ = – 0.0523; *p*_+_ = 0.5720; point estimate

a *L*_C_/*L*_τ_ are the one-sided 95% lower error-reduction margins from 5000 paired circular moving-block draws (400 frames, 4 ps), and *p*_+_ is the fraction of draws with both gains positive. The resampling measures finite-trajectory sensitivity, not independent molecular replication.

### 4.5 Cost and Scalar-Transfer Comparison

The C6-calibrated scalar factor was transferred unchanged to the other three coordinates. It improved both errors in three of four point-estimate comparisons because it improved C5 relaxation while worsening the C5 curve; the finite-history or surrogate candidate improved both errors in all four (Table 8). Its mean joint gain was 10.88% versus 3.91% for scalar rescaling, and its worst joint margin was 1.93% versus −11.53%, at the higher fitting and trajectory validation cost reported in the table. Acetone retained a larger scalar relaxation gain, so the comparison does not show uniform superiority.

**Table 8:** Post-Decision Scalar-Transfer and Cost Comparison^a^

coordinate	scalar factor 0.855 *G*_C_/*G*_τ_/*J* (%)	finite history/surrogate *G*_C_/*G*_τ_/*J* (%)	comparison
C6 end-to-end	13.37/33.09/13.37	23.95/43.57/23.95	Both pass; finite history larger
C5 end-to-end	–11.53/92.58/–11.53	33.97/4.01/4.01	Scalar harms curve; finite history passes
toluene ring-methyl	4.99/1.56/1.56	51.53/1.93/1.93	Both pass; finite history larger
acetone methyl–methyl	12.24/94.79/12.24	13.62/73.49/13.62	Scalar has larger *G*_τ_; surrogate larger *J*
four-coordinate summary	both errors improve in 3/4 point estimates; mean/best/minimum *J* = 3.91/13.37/–11.53%	both errors improve in 4/4 point estimates; mean/best/minimum *J* = 10.88/23.95/1.93%	2.8 × mean joint gain at higher validation cost; uncertainty reported by experiment

a Entries are *G*_C_/*G*_τ_/*J*, where *J* = min­(*G*_C_, *G*_τ_). PMF-only CG is the 0/0/0% reference. The scalar method uses a 1501-point C6 scan and no corrected rollout; the finite-history/surrogate method uses 21000 pretest rows and 7200 held-out generated/scored frames.

The lower-bound deployment decisions for the supporting 13-coordinate breadth panel are summarized in Figure 3.

**Figure 3:**
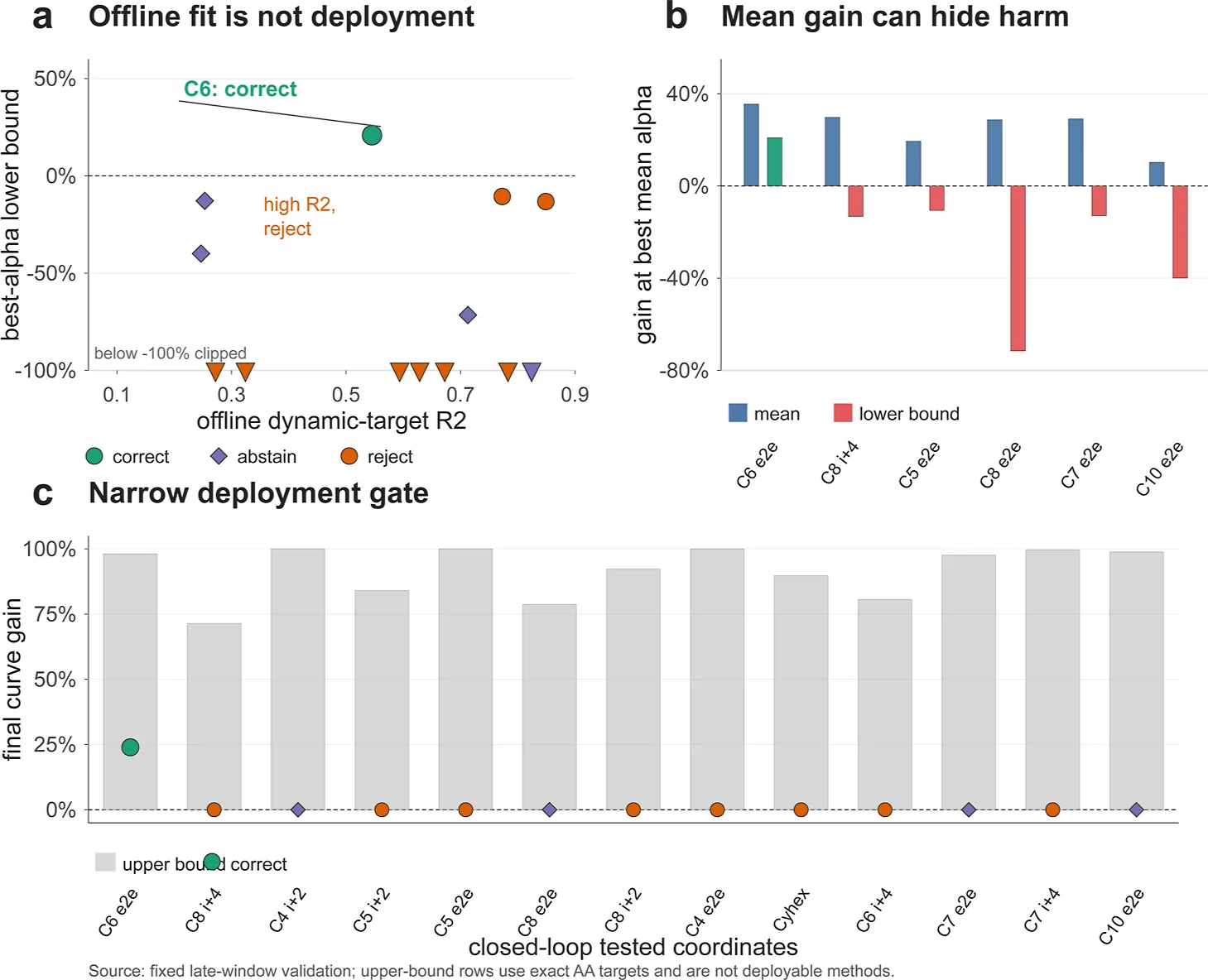
Lower-bound deployment decisions for the supporting 13-coordinate breadth panel. Panel (a) plots offline dynamic-target *R* ^2^ against the minimum held-out lower bound at the best tested nonzero strength; only C6 is above zero. Panel (b) contrasts mean gain with its lower bound and shows why a positive mean alone does not authorize deployment. Panel (c) compares the nondeployable AA-target upper-bound curve gain (gray bars) with the final correct, abstain, or reject decision. Thus, offline fit and target-informed recoverability are diagnostic, whereas deployment requires the generated-trajectory lower-bound criterion.

### 4.6 Independent Surrogate Comparison

The distinct OpenFF/OpenMM AR(2) surrogate improved both point estimate errors for two compact molecules, ibuprofen and cholesterol (Table 9; Figure 4). It exceeded the separately fitted scalar joint gains in all four comparisons. Cholesterol remained a boundary case because substantial relaxation time error persisted despite improvement over the PMF-only process.

**Table 9:** OpenFF/OpenMM Stochastic-Surrogate Comparison of the Shared Criterion^a^

coordinate(full/retained atoms)	PMF-only error	scalar *J* (%)	surrogate result	interpretation
acetone methyl–methyl (10/4)	1.07/581.99%	6.82	*J* = 9.89%; final *E*_τ_ = 0.59%	both point estimates improve; compact carbonyl
toluene methyl–ring centroid (15/7)	1.05/606.20%	5.71	*J* = 8.24%; final *E*_τ_ = 1.00%	both point estimates improve; aromatic
ibuprofen carboxyl–isobutyl centroid (33/15)	1.35/632.83%	23.73	*J* = 42.11%; final *E*_τ_ = 28.07%	both point estimates improve; flexible drug-like
cholesterol hydroxyl–tail centroid (74/28)	2.14/878.83%	39.10	*J* = 43.43%; final *E*_τ_ = 204.50%	both point estimates improve; incomplete slow recovery

a Each AR(2) surrogate uses 10,000 fitting and 10,000 held-out frames. PMF-only error is reported as curve NRMSE/relaxation-time relative error; scalar and surrogate entries are joint gain *J.*

**Figure 4:**
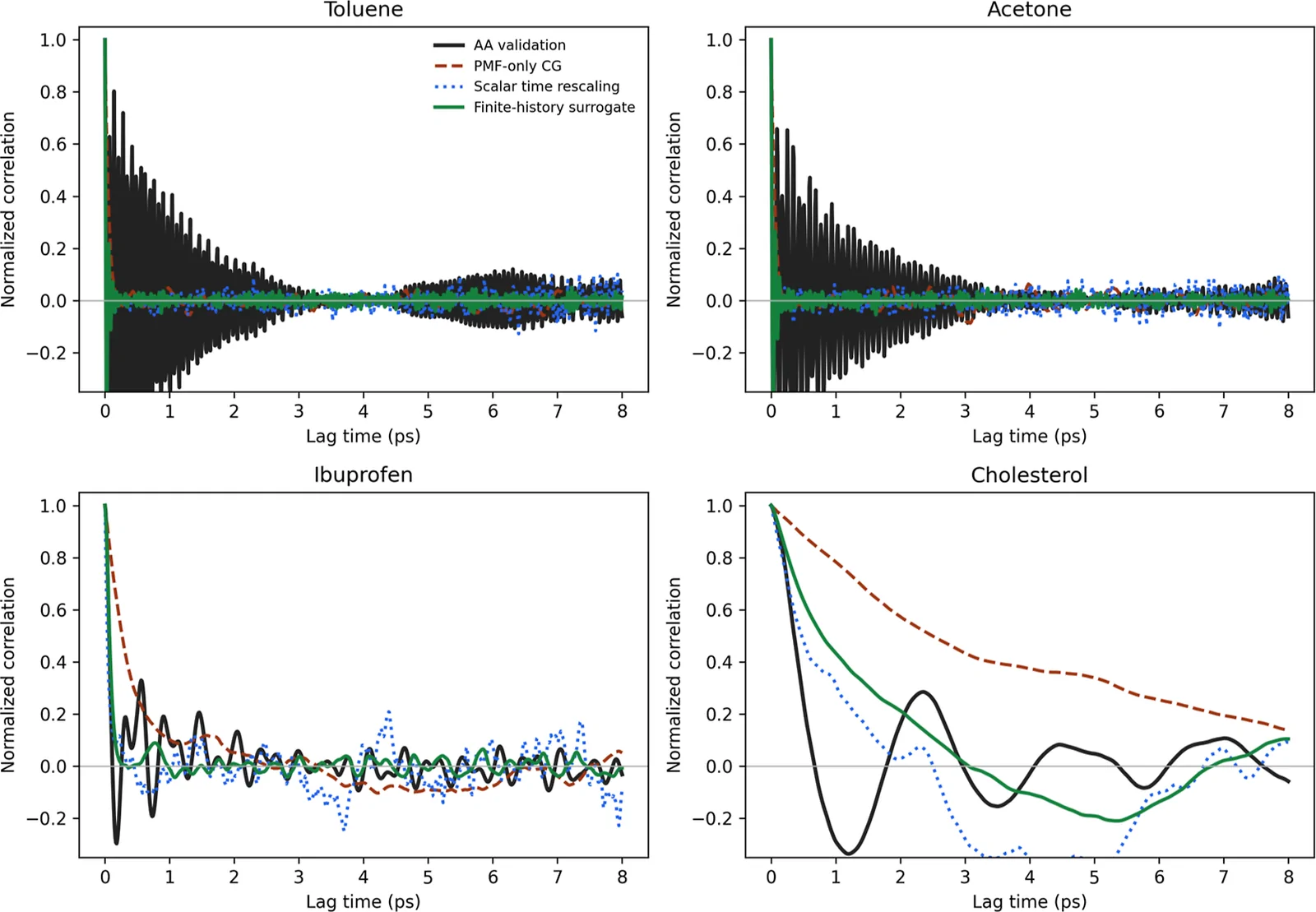
OpenFF/OpenMM finite-history surrogate comparison panel. The finite-history-labeled curve is the stochastic AR(2) process in eq 13, not the additive kinematic/QVA update of the complete PMF-baseline experiments. Acetone, toluene, ibuprofen, and cholesterol are scored on the withheld halves of their OpenFF/OpenMM coordinate trajectories. Both point-estimate errors improve for all four; cholesterol also shows that improvement need not imply complete relaxation-time recovery.

### 4.7 Diagnostic Boundary Inventory

The 45-coordinate inventory assigned 33 coordinates to local short memory, eight to finite kernels, one to state conditioning, and three to abstention or richer hidden treatment (Table 5). These are diagnostic assignments, not deployments; row-level definitions are indexed in Supporting Information, Section S5.

## 5 Discussion

### 5.1 Overall Result Pattern

Tables 1–9 contain the numerical record by experiment. The complete C6 and C5 comparisons used one fitted PMF process for the uncorrected comparator, correction target, and corrected process; both met the structural tolerance and had positive one-sided lower bounds for both dynamical error reductions. Applicability diagnosis stopped acetone C–O before a richer state or hidden-variable treatment and ethanol heavy-atom end-to-end before a finite-memory treatment. The 13-coordinate breadth panel returned one nonzero result, four zero-strength outcomes, and eight rejections. Diagnosis-informed tests added C5, toluene, alanine, and the predeclared acetone surrogate, with both paired-block lower margins positive for C5 and toluene. In the cost comparison, the finite-history or surrogate correction improved both point-estimate errors in four comparisons versus three for the C6-calibrated scalar clock and increased the mean joint gain by *a* factor of 2.8. The separate OpenFF/OpenMM AR(2) arm improved both point-estimate errors on four coordinates, with cholesterol retaining substantial slow-coordinate error.

### 5.2 Alignment of the Experimental Design, Results, and Method

The abstract method requires a PMF-referenced error object, a correction fitted only when the residual-memory diagnosis supports its feature family, and a final comparison of uncorrected and corrected generated trajectories. The C6 and C5 experiments instantiate those requirements: one detailed-balance PMF transition defines the baseline mean increment in eq 4, receives the fitted increment in eq 6, and generates both paired trajectories. Disjoint fit, selection, and final blocks with newly seeded final replicas test the correction rather than its fitting accuracy. Acetone C–O and ethanol heavy-atom end-to-end instantiate the preceding applicability step; stopping before a nonzero fit is the specified result when diagnosis selects a richer model. The zero-residual 13-coordinate analysis and AA-target upper bound are therefore limited to breadth, selectivity, and recoverability.

Within the declared coordinates and windows, C6 and C5 met the structural criterion and improved both dynamic errors; larger strengths were excluded when they crossed the PMF tolerance. The two applicability decisions likewise withheld the low-cost correction when the memory class was inadequate. High offline *R* ^2^, large AA-target upper-bound gains, or curve improvement alone did not ensure a safe-generated trajectory. Added fitting and validation changed the harmful worst scalar margin to a positive one, although scalar rescaling retained the larger acetone relaxation time gain. The OpenFF/OpenMM arm tests the same two-observable decision criterion through a distinct AR(2) surrogate, not numerical transfer of the kinematic/QVA update. These results support conditional correction, not the universal superiority or equivalence of the implementations.

### 5.3 Subproblems and Limits

The first subproblem is whether the post-PMF error can be stated as an observable projected memory problem rather than a generic residual force score. The qualified PMF-only discrepancies, recoverability bounds, and 45-coordinate inventory answer this operationally: 42 coordinates received an explicit memory class, and three were withheld from low-cost treatment. This identifies a finite window target and candidate class without claiming a unique microscopic cause or exact orthogonal dynamics.

The second subproblem is the no-harm correction of that target. In the complete C6/C5 experiments, one PMF transition received the retained increment, stayed within the structural tolerance, and improved both dynamical errors on the final stochastic replicas. The structural veto at strength 0.25 shows why dynamics alone are insufficient, while the two precorrection stops show why a nonzero candidate is withheld from an inadequate memory class. The C5 breadth panel candidate failed, whereas the diagnosis-informed QVA-plus-signed candidate passed; these candidate-specific outcomes are not contradictory. The method therefore returns correct, abstain, reject, or escalate without assuming that every diagnosed coordinate has a low cost solution. Evidence strength also remains tiered: both paired-block lower margins are positive only for C5 and toluene, while alanine and acetone remain point estimate results.

The third subproblem is whether this selectivity has practical value and transfers beyond one controlled panel. Relative to the unchanged C6-calibrated clock, the finite-history candidate changed the worst joint margin from −11.53 to 1.93% and increased the mean joint gain from 3.91 to 10.88%, at the stated cost of 21,000 pretest rows and 7200 held-out generated/scored frames. The OpenFF/OpenMM arm extends the same two-observable no-harm criterion to compact carbonyl and aromatic coordinates, a flexible drug-like coordinate, and a larger steroid-like coordinate; cholesterol shows that no-harm improvement need not mean complete relaxation recovery.

A full GLE, finite-kernel, state-conditioned, or hidden-state model can represent more memory channels than the low-cost candidates tested here, and several rejected coordinates may require such models. The present method is a prior deployment screen and not a replacement for those representations. Its scope is limited to the reported united-atom and molecular distance coordinates and response windows; the OpenFF/OpenMM arm is a gas-phase transfer test, not a full solvent, polymer, force field family, or long time scale benchmark. For a new coordinate, observable, force field, or window, the PMF-only dynamic error, memory class, candidate, and held-out no-harm decision must be qualified again.

## 6 Conclusions

We treated the component of finite-window observable error supported by residual-memory diagnostics as a missing-friction target in PMF-preserving molecular CG models. The resulting bounded deployment problem asks whether enough of the diagnosed response can be recovered to improve the declared observables without reconstructing every lost force or sacrificing the efficiency of coarse-graining.

In the complete C6 and C5 experiments, uncorrected and corrected replicas shared one detailed balance PMF process on untouched final blocks. Corrected PMF L1 distances were 0.05401 and 0.06071, below the 0.10 limit, and the one-sided 5% lower bounds of both dynamic error reductions were positive. Larger strengths were withheld when they crossed the structural limit despite favorable dynamic margins, so both structure and dynamics were tested on the corrected process itself.

The same method stopped before correction when its low-cost assumptions were not met. Acetone C–O required a richer state or hidden-variable description, and ethanol heavy-atom end-to-end required a finite-memory kernel; neither case received a nonzero fit or corrected trajectory. Supporting breadth and selectivity evidence gave the 13-coordinate breadth-panel outcome of one nonzero C6 correction, four abstentions, and eight rejections. Diagnosis- informed reruns supported C5, toluene, alanine, and the predeclared acetone surrogate, while direct operator checks and the 45-coordinate inventory supported the preceding applicability diagnoses.

Against the C6-calibrated scalar clock, the finite-history or surrogate correction improved both point-estimate errors in 4/4 comparisons versus 3/4, increased mean joint gain from 3.91 to 10.88%, raised the best gain from 13.37 to 23.95%, and moved the worst margin from −11.53 to 1.93%. These gains required 21,000 pretest rows and 7200 held-out generated/scored frames across four coordinates; uncertainty is reported separately for each experiment.

The distinct OpenFF/OpenMM two-lag surrogate tested the shared decision criterion without serving as a numerical transfer of the additive implementation. It improved both point estimate errors on acetone, toluene, ibuprofen, and cholesterol, with joint gains from 8.24 to 43.43%. Cholesterol retained an incomplete slow-relaxation recovery.

The resulting contribution is a reproducible conditional decision standard for missing friction correction. It links the observable projected-memory object, memory-class diagnosis, generated-trajectory evidence, correction cost, and deployment decision. It corrects only after PMF transfer, applicable-class and nonzero-candidate selection, corrected-process structural preservation, and held-out dynamical acceptance; otherwise, it abstains, rejects, or escalates. Broader solvent, polymer, force-field family, and full hidden-state mechanisms remain future tests. Within the tested scope, the rule recovers a useful part of the diagnosed response without making CG correction open-ended.

## Supplementary Material





## Data Availability

The accompanying code/data ZIP provides the machine-readable inputs, result tables, analysis and figure-generation code, task registry, environment specifications, SHA-256 manifests, and verification scripts used for the reported results. Its README and task registry give the command, expected check, and scope of each task. Source-generation tasks verify protocol execution rather than bitwise trajectory identity, whereas fixed-input tasks verify the reported numerical values.

## Notes

Coordinate and site-index definitions; source and reduced-dynamics protocols; PMF estimation; finite-history features and rollout settings; complete panel results; and verification instructions (PDF)
Machine-readable inputs and result tables; analysis and figure-generation code; task registry; environment specifications; SHA-256 manifests; and verification scripts (ZIP)
