# Supplementary material for: No-Harm Missing-Friction Correction for Potential-of-Mean-Force-Matched Coarse Graining
Source: ACS Omega. 2026 Aug 19;11(34):52029–40. doi: 10.1021/acsomega.6c07213 (PMC13625116; doi:10.1021/acsomega.6c07213)
Supplement: Supplementary file 1 [file ao6c07213_si_001.pdf]

Supporting Information

No-Harm Missing-Friction Correction for Potential-of-Mean-Force-Matched Coarse Graining

Chunlin Zhao,† Liwen Liu,† Dongshi Zuo,† Rigeng Wu,† Liqian Zhang,† Yongan Zhang,† Yingchun Guo,† and Meian Li*,†,‡

†College of Computer and Information Engineering, Inner Mongolia Agricultural University, Hohhot 010018, China

‡Inner Mongolia Key Laboratory of Big Data Research and Application in Agriculture and Animal Husbandry, Inner Mongolia Agricultural University, Hohhot 010018, China

*E-mail: limeian1973@126.com

S1. Purpose and evidence roles

This Supporting Information contains the definitions, numerical protocol, and artifact map needed to reconstruct the main manuscript. It does not introduce a second method. The scientific chain is

$$\begin{aligned}\text{AA trajectory} &\rightarrow \text{retained coordinate, PMF qualification, and panel-specific uncorrected process}\\ &\rightarrow \text{finite-window post-PMF observable error}\\ &\rightarrow \text{observable projected-memory object}\\ &\rightarrow \text{declared finite-history projection and rollout}\\ &\rightarrow \text{four-action held-out no-harm decision}\\ &\rightarrow \text{same-data cost comparison and unresolved remainder.}\end{aligned}$$

Here AA is the higher-resolution-reference label used in the equations and tables; for the linear-alkane panel, that source is explicitly the stated united-atom model. CG denotes coarse grained, and PMF denotes potential of mean force.

The evidence has distinct, noninterchangeable roles. The complete C6/C5 experiments use the same PMF process to define the uncorrected comparator, correction target, and corrected process. Acetone C–O and ethanol heavy-atom end-to-end test residual-memory applicability before a nonzero correction is fitted. The 13-coordinate panel and targeted molecular-coordinate tests provide supporting breadth, selectivity, and additional generated-trajectory results. The four-molecule OpenFF/OpenMM calculation tests the shared criterion with an archived stochastic AR(2) implementation that is distinct from the additive kinematic/QVA update. These result sets are reported according to their roles and are not pooled numerically.

S2. Notation, units, and model identity

Table S1: Notation used in the main text and Supporting Information.

Symbol	Definition	Unit or range
$\mathbf{r}_t, \mathbf{v}_t$	Full source-model site positions and velocities	nm; nm ps^{-1}
$Q_t = \xi(\mathbf{r}_t)$	Retained random distance coordinate	nm
q	A scalar value of Q	nm
$T, w(t)$	Response-window length and uniform normalized weight, $w(t) = 1/T$ on $[0, T]$ and zero outside	ps; ps^{-1}
m_Q	Retained-coordinate mass	Da
U_{PMF}	Training-density potential of mean force	kJ mol^{-1}
$\zeta, \delta\zeta$	Eliminated bath variable and its conditional centering at fixed q	formal bath variables
$C_q^\zeta(t), S_q$	Conditional bath correlation and its time integral	correlation; integrated transport
$A(q)$	Local map from centered bath variation to unresolved force along Q	force per bath unit
$S_q^0, \Delta S_q$	Transport represented by the declared baseline and the effective missing part	integrated transport
$\Pi_{\mathrm{obs}}, \mathcal{O}_Q$	Restriction to the declared response and the observable projected-memory object	response operator
$\mathcal{H}, \phi_{\mathcal{H}}, \theta$	Declared finite-history family, feature vector, and training-fitted coefficient vector	feature-model objects
λ	Ridge regularization coefficient in the finite-history fit; fixed at 10^{-3}	dimensionless
$\widehat{\Delta}_0, \mathcal{C}_{\mathrm{tr}}$	Fitted baseline increment and training-fixed stability clip	coordinate increment; map
α	Dimensionless correction strength; candidate sets are model specific	see text below
QVA	Coordinate–velocity–acceleration finite-history feature family; abbreviation used in the data archive	feature-family label
$\mathcal{M}_0, \mathcal{M}_{\mathrm{cand}}$	Panel-specific uncorrected and admissible candidate reduced processes	process labels
$C(t)$	Normalized coordinate autocorrelation	dimensionless
τ	Integral of the first positive lobe of $C(t)$	ps
E_C, E_τ	Correlation-curve and relaxation-time errors	nonnegative
G_C, G_τ	Fractional error reductions relative to the panel-specific uncorrected process	dimensionless

Source dynamics and projected-memory construction. Let $\mathbf{r}_a$ and $\mathbf{v}_a$ denote the positions and velocities in the higher-resolution source model, labeled AA in the manuscript. The source trajectories obey

$$\mathrm{d}\mathbf{r}_a = \mathbf{v}_a\,\mathrm{d}t, \tag{S1}$$

$$m_a\,\mathrm{d}\mathbf{v}_a = -\nabla_a U(\mathbf{r})\,\mathrm{d}t - m_a\gamma_{\mathrm{AA}}\mathbf{v}_a\,\mathrm{d}t + \sqrt{2m_a\gamma_{\mathrm{AA}}k_{\mathrm{B}}T}\,\mathrm{d}\mathbf{W}_a, \tag{S2}$$

with independent Cartesian Wiener processes. The retained random coordinate is $Q_t = \xi(\mathbf{r}_t)$; lowercase q denotes one scalar value of that coordinate. For a site-pair distance,

$$Q = \|\mathbf{r}_j - \mathbf{r}_i\|, \qquad \dot{Q} = \mathbf{e}\cdot(\mathbf{v}_j - \mathbf{v}_i), \qquad \mathbf{e} = \frac{\mathbf{r}_j - \mathbf{r}_i}{Q}. \tag{S3}$$

At fixed q, let ζ_t be the eliminated bath variable with conditional equilibrium measure μ_q, and write $\delta\zeta_t = \zeta_t - \langle\zeta\rangle_{\mu_q}$. After PMF subtraction, the locally visible unresolved force is represented to first order as $F_\perp(q,\zeta) \simeq A(q)\delta\zeta$. For an integrable conditional bath correlation,

$$C_q^\zeta(t) = \mathbb{E}_{\mu_q}[\delta\zeta_t \otimes \delta\zeta_0], \qquad S_q = \int_0^\infty C_q^\zeta(t)\,\mathrm{d}t = \langle\delta\zeta, \chi_q\rangle_{\mu_q}, \qquad -\mathcal{L}_q\chi_q = \delta\zeta. \tag{S4}$$

Here $\mathcal{L}_q$ is the conditional bath generator and χ_q is the centered Poisson corrector. The correlation integral and Poisson representation are two forms of the same transport object. If S_q^0 denotes the part already represented by the declared PMF-only process, then $\Delta S_q = S_q - S_q^0$. Observable projection gives the main-text object $\mathcal{O}_Q = \Pi_{\mathrm{obs}}[\beta A(q)\Delta S_q A(q)^\mathsf{T}]$. The calculation does not estimate the full tensors separately; it approximates their consequence for the declared coordinate response by the finite-history increment defined below.

Memory class is the diagnostic assignment of the unresolved response; feature family $\mathcal{H}$ is the numerical span used to construct a candidate. A diagnosed class determines which family may be tested, but the two labels are not interchangeable.

Memory-class-matched finite-history runs use

$$\alpha \in \{0, 0.1, 0.25, 0.5, 0.75, 1\}.$$

The 13-coordinate C6 result retains its independently fixed value $\alpha = 0.45$. The candidate set is therefore part of each declared finite-history calculation, not a global physical parameter.

S3. 13-coordinate breadth panel

The panel contains 12 distances in custom united-atom linear alkanes and one exploratory cyclohexane distance. C5 and C6 identify five- and six-site linear alkanes; their end-to-end coordinates use zero-based terminal pairs 0–4 and 0–5. Every internal label denotes one fixed pair, not an average over possible values of i; the questioned C8 $i, i+4$ label is the zero-based pair 1–5. The force-field and trajectory details are recorded here.

For the linear alkanes, terminal and interior site masses are 15.035 and 14.027 amu. Harmonic bonds use $r_0 = 0.153$ nm and $k_r = 224262$ kJ mol^{-1} nm^{-2}; harmonic angles use $\theta_0 = 112.7°$ and $k_\theta = 488.3$ kJ mol^{-1} rad^{-2}. The three periodic dihedral terms have periodicities 1, 2, and 3 and force constants 5.9, 1.1, and 13.2 kJ mol^{-1}. Uncut Lennard–Jones interactions use $(\sigma, \epsilon) = (0.375\,\mathrm{nm}, 0.8148\,\mathrm{kJ\,mol^{-1}})$ for terminal sites and $(0.395\,\mathrm{nm}, 0.3824\,\mathrm{kJ\,mol^{-1}})$ for interior sites; 1–2 and 1–3 pairs are excluded. OpenMM Langevin-middle dynamics were run at 300 K with damping 1 ps^{-1} and a 1 fs step. Row-level lengths, strides, seeds, and platforms are in the machine-readable registry. The exploratory cyclohexane model contains bonded, Lennard–Jones, and charge terms and was sampled at 300 K with damping 2 ps^{-1}, a 0.5 fs step, 20000 equilibration steps, and 200000 production steps saved every 20 steps. It is used only as a boundary comparison, not as transferable force-field evidence.

For every row, only the archived AA coordinate was admitted as source data. The PMF was refit with the protocol in Section S4, and a new PMF-only trajectory was generated. Previously generated reduced-coordinate arrays and learned corrections were not reused. The source archive has SHA-256 `0027e23cb25303ecce063703d7c0b871822906be2398268eaaf363b4f967024f`; the inventory records the hash, frame count, array keys, and evidence role of each of the 13 extracted NPZ files.

Table S2: Uniform PMF-only analysis of the 13-coordinate breadth panel. Static L1 is calculated from the pooled eight-replica distribution on the PMF grid. The last two columns are baseline errors, not correction gains.

Coordinate	AA frames	Static L1	E_C	E_τ
C6 e2e	10000	0.073	0.990	0.888
C8 $i, i+4$	50000	0.036	0.943	0.747
C4 $i, i+2$	50000	0.036	0.967	0.531
C5 $i, i+2$	50000	0.037	0.986	0.663
C5 e2e	50000	0.029	0.991	0.996
C8 e2e	50000	0.034	0.964	0.892
C8 $i, i+2$	50000	0.042	0.927	0.569
C4 e2e	50000	0.034	0.990	0.816
Cyclohexane	10000	0.079	0.946	0.454
C6 $i, i+4$	10000	0.069	0.994	0.994
C7 e2e	10000	0.065	0.961	0.877
C7 $i, i+4$	10000	0.074	0.977	0.890
C10 e2e	10000	0.077	0.985	0.946

The archived force array is only the Cartesian potential force projected along the distance gradient. Because velocities were not saved, it is not treated as a complete radial-force record. The breadth panel is used for the declared PMF-only and finite-history calculations only.

S4. PMF baseline, finite-history implementations, and comparisons

PMF fitting and 13-coordinate baseline. The PMF support is the interval between the 0.001 and 0.999 training quantiles, padded by 10% of their separation on each side. A 160-bin histogram receives a 0.5-count pseudocount and Gaussian smoothing of width 1.5 bins with nearest boundary extension. If p_i is the resulting normalized bin mass, then $u_i = U_i/(k_BT) = -\log p_i + \text{constant}$.

For the 13-coordinate PMF-transfer baseline and the complete C6/C5 experiments, the overdamped Smoluchowski equation is discretized on the same grid. For neighboring bins i and j separated by h, the rate is

$$k_{ij} = \frac{D}{h^2}\exp[-(u_j - u_i)/2], \qquad D = \frac{k_BT}{\gamma}.$$

Here $\gamma = \mu\gamma_{AA}$ is fixed by the reduced mass and the declared source-model damping rate: $\gamma_{AA} = 1\ \text{ps}^{-1}$ for the linear-alkane rows and $2\ \text{ps}^{-1}$ for cyclohexane. This parameter belongs to the prespecified PMF-only breadth baseline; it is not estimated from the observed dynamical mismatch. No rate leaves the support, which gives a reflecting boundary. The diagonal entry is minus the sum of outgoing rates. The transition over the 0.01 ps output interval is the matrix exponential of this generator. Before use, the verification script checks row stochasticity, stationarity of $\pi_i \propto e^{-u_i}$, and detailed balance $\pi_i P_{ij} = \pi_j P_{ji}$. The pooled fixed-grid histogram L1 qualification limit is 0.10. For normalized histogram masses, L1 distance is twice total-variation distance, so this limit bounds the PMF-coordinate distribution discrepancy by 0.05 in total variation. It qualifies the baseline only and is not used to select a correction family, coefficient, or strength in the 13-coordinate breadth

panel. In the complete C6/C5 experiments, the same limit is applied to both baseline and corrected replica pools as a structural eligibility criterion.

Finite-history correction. For the method, the training target is the AA one-step increment minus the conditional mean increment of the declared baseline process. In the complete C6/C5 experiments that baseline is the fitted PMF transition; the original 13-coordinate analysis uses its zero-residual discrete comparator. A ridge model projects that target onto the declared kinematic, QVA, or signed-memory feature family, and the scaled update is added recursively to the generated baseline trajectory. The family, coefficient, scale ladder, and stability clip are held fixed before held-out scoring. These are generated correction rollouts judged by the two-observable no-harm criterion, not offline predictability screens.

Exact zero-residual breadth implementation. For the original 13-coordinate breadth analysis, let R_n be the archived training residual-force channel and define

$$\mathbf{x}_n(R_n) = (1, Q_n, v_n, a_n, \overline{F}(Q_n), R_n)^{\mathsf{T}}, \qquad \Delta Q_n = Q_{n+1} - Q_n, \tag{S5}$$

with nonintercept features standardized using training means and standard deviations. The transition coefficients are fitted only on training rows,

$$\widehat{\beta}_0 = \arg\min_{\beta} \sum_{n\in\text{train}} [\Delta Q_n - \beta^{\mathsf{T}}\widetilde{\mathbf{x}}_n(R_n)]^2 + \lambda_0\|\beta_{1:}\|_2^2, \qquad \lambda_0 = 10^{-3}. \tag{S6}$$

The uncorrected breadth comparator is the zero-residual member

$$\widehat{\Delta}_0(\mathsf{H}_n) = \mathcal{C}_{\Delta,\text{tr}}[\widehat{\beta}_0^{\mathsf{T}}\widetilde{\mathbf{x}}_n(0)], \tag{S7}$$

where the increment clip is fixed by the 0.001/0.999 training quantiles. The finite-history target and ridge fit are

$$y_{n+1} = (Q_{n+1}^{\text{AA}} - Q_n^{\text{AA}}) - \widehat{\Delta}_0(\mathsf{H}_n), \tag{S8}$$

$$\widehat{\theta} = \arg\min_{\theta} \sum_{n\in\text{train}} [y_{n+1} - \theta^{\mathsf{T}}\phi_{\mathcal{H}}(\mathsf{H}_n)]^2 + \lambda\|\theta\|_2^2. \tag{S9}$$

With training-fixed scaling and clipping, the generated corrected trajectory is

$$Q_{n+1}^{(\alpha)} = \mathcal{C}_{\text{tr}}\left[Q_n^{(\alpha)} + \widehat{\Delta}_0(\mathsf{H}_n^{(\alpha)}) + \alpha\widehat{\theta}^{\mathsf{T}}\phi_{\mathcal{H}}(\mathsf{H}_n^{(\alpha)})\right]. \tag{S10}$$

This comparator and rollout support the original breadth and selectivity analysis. They are distinct from the complete C6/C5 same-PMF experiments, where one PMF transition defines the target, uncorrected process, and corrected process.

The primary 13-coordinate calculation used 6000 pretest frames per coordinate. Fitting and internal validation windows were both contained within those 6000 frames. The remaining 1208 rows formed a held-out continuation; the fixed 1200-frame rollout cap set the number of generated and scored frames. The AA-target upper bound is a separate, nondeployable diagnostic: at each held-out step it inserts the exact contemporaneous AA-minus-zero-residual-baseline increment target in place of a predicted correction, under the same recursive propagation and clipping. It therefore measures target-informed recoverability, not a learned correction, causal identification, or deployability.

Complete PMF-baseline correction experiments. The complete experiments used C6 end-to-end and C5 end-to-end. The PMF-transition conditional mean increment was subtracted from the AA increment on the correction-fit block, after which the retained kinematic-plus-signed (C6) or QVA-plus-signed (C5) family was fitted. Baseline and corrected replicas used the same detailed-balance transition, invariant initial-state law, reflecting support, burn-in, and uniform draws. The four data roles and index ranges were

$$\begin{aligned} \text{C6: PMF fit } [0, 4000), &\quad \text{correction fit } [4000, 6000), \\ \text{validation } [6000, 8000), &\quad \text{final test } [8000, 10000); \end{aligned}$$

$$\begin{aligned} \text{C5: PMF fit } [0, 20000), &\quad \text{correction fit } [20000, 25000), \\ \text{validation } [25000, 37500), &\quad \text{final test } [37500, 50000). \end{aligned}$$

Signed-memory and feature quantities used only the PMF-fit and correction-fit blocks.

The validation grid was $\{0.1, 0.25, 0.5, 0.75, 1\}$. Each candidate used 12 paired validation replicas; the passing candidate maximizing the smaller dynamical lower bound was fixed. Final scoring used 24 newly seeded pairs, a 20 ps burn-in, and 5000 paired bootstrap draws. Both baseline and corrected pooled histograms had to satisfy PMF L1 ≤ 0.10, and the one-sided 5% lower bounds of both absolute dynamical error reductions had to be positive. The L1 limit is equivalent to total-variation distance ≤ 0.05, so any coordinate observable scaled to [0,1] has a generated-versus-fitted-PMF expectation discrepancy no greater than 0.05. This stochastic-replica bootstrap does not represent independent molecular replication.

Pre-correction applicability calculations. For acetone C–O and ethanol heavy-atom end-to-end, the released atomistic coordinate and projected-force arrays define $F_\perp = F_Q - \mathbb{E}[F_Q \mid Q]$. The positive-area residual-force correlation time τ_F^+ and the coordinate correlation time τ_Q give $\epsilon_{\text{sep}} = \tau_F^+/|\tau_Q|$. ϵ_{state} is the interquartile range divided by the median of τ_F^+ across five equal-population coordinate bins. The prespecified short-memory limits are $\epsilon_{\text{sep}} \leq 0.35$ and $\epsilon_{\text{state}} \leq 1.0$; a finite-kernel assignment permits $\epsilon_{\text{sep}} \leq 1.0$. These values are the same routing settings recorded in the submitted `theory_boundary_validation_20260616.json` and the corresponding script defaults; they were not estimated from the added correction outcomes. The 0.35 cutoff denotes a positive force-memory time no larger than roughly one third of the coordinate time, the 1.0 cutoff marks equal force and coordinate time scales, and the state cutoff requires the across-bin interquartile range not to exceed the median. They define the tested model classes rather than universal physical constants. Acetone C–O gave 2.773 and 5.367 and was assigned to richer state or hidden variables. Ethanol heavy-atom end-to-end gave $\epsilon_{\text{sep}} = 0.890$ and was assigned to a finite-memory kernel. Both calculations ended with $\alpha = 0$ before correction fitting; neither produced a corrected trajectory.

No-harm acceptance means that both declared dynamical errors improve and every predeclared stability window remains finite and nondivergent. A nonzero candidate that passes held-out scoring is *correct*; absence of a safe nonzero candidate without a hard-safety veto is *abstain*; a declared validation-stage hard-safety veto or failure of held-out acceptance is *reject*; and failure of the declared low-cost memory class is *escalate*. Thus C4 $i, i+2$ has final action *abstain*, while its harmful best nonzero candidate is *rejected* and retained only as diagnostic boundary evidence; C8 $i, i+4$ is *reject* because the validation selector recorded an unstable-response-island veto.

Table S3: Exact diagnostic families, generated-rollout candidates, and fixed settings.

Calculation	Feature vector or setting	Fixed value / source
13-coordinate diagnostic screen	QVA; v-history L4; Q-history L4; joint Q/v-history L4; quadratic QVA	`dynamic_target_mechanism_discriminator_20260616.py`
13-coordinate zero baseline	No correction	`B0_zero`
13-coordinate v-history candidate	(v_0, v_1, v_2, v_3)	`B2_memory_L4`
13-coordinate Q-history candidate	$(Q_n, Q_{n-1}, Q_{n-2}, Q_{n-3})$	`B2_q_history_L4`
13-coordinate joint-history candidate	$(Q_n, \ldots, Q_{n-3}, v_0, \ldots, v_3)$	`B2_qv_history_L4`; Figure 3 candidate
13-coordinate upper bound	Exact contemporaneous AA-minus-zero-residual-baseline increment	`oracle_delta`; nondeployable
13-coordinate alpha grid	$\{0, 0.01, \ldots, 0.10,$ $0.12, 0.15, 0.18, 0.20, 0.25,$ $0.30, 0.35, 0.40, 0.45, 0.50\}$	Fixed original driver
Diagnosis-informed kinematic/signed	$(v_0, v_1, v_2, v_3, m_\Sigma, m_{\cos}, m_{\sin}, m_{\exp})$	`operator_guided_error_predictor_closed_loop_20260617.py`
Diagnosis-informed QVA/signed	$(Q_n, v_0, a_0, \overline{F}, m_\Sigma, m_{\cos}, m_{\sin}, m_{\exp})$	Same source and target
Targeted-run numerical settings	Pretest rows; minimum history; memory horizon; ridge; clips; candidate strengths	5000; 16 frames; 2 ps; 10^{-3}; 0.001/0.999 quantiles; $\{0, 0.1, 0.25, 0.5, 0.75, 1\}$
Accepted fixed strengths	C6; C5; toluene; alanine; acetone surrogate	0.45; 0.1; 0.5; 1.0; 0.5
Complete PMF-baseline experiments	C6 kinematic-plus-signed; C5 QVA-plus-signed; shared PMF transition component	12 validation + 24 final replica pairs; 5000 bootstrap draws; selected $\alpha = 0.10$ for both

The five-family diagnostic screen is an offline target-predictability test; it must not be substituted for the generated-rollout candidate set. The signed-memory quantities are the causal velocity convolutions defined in the main text. All feature centering/scaling, state edges, kernel parameters, and clips are estimated from training rows only. For toluene ring–methyl, the inventory label `state_conditioned_operator` records the state-sensitive diagnostic class. The accepted correction itself is the kinematic-plus-signed finite-history vector in Table S3; it contains no state indicator and is not a state-conditioned propagator.

Fixed validation selector. The targeted validation screen used training fractions 0.36, 0.42, 0.48, and 0.54, with at most 800 generated frames per window. A primary window required curve gain at least 0.05, tau gain at least -0.10, and pointwise gain at least -0.10; curve or pointwise worsening greater than 0.25 triggered the hard-safety rejection. Let N_p and N_c denote the numbers of primary and hard-safety windows; overbars and the superscript min denote the mean and minimum validation-window gains. The default selector preferred the 0.25 strength on ties and required

$$N_p \geq 1, \quad N_c = 0, \quad \overline{G}_C \geq 0.02, \quad G_C^{\min} \geq -0.02, \quad G_\tau^{\min} \geq -1.00, \quad G_{\text{point}}^{\min} \geq -0.20.$$

The fixed overrides were as follows. The richer-hidden class allowed QVA/signed candidates with $\alpha \leq 0.5$, $N_p \geq 2$, $N_c = 0$, $\overline{G}_C \geq 0.25$, $G_C^{\min} \geq 0$, and $G_{\text{point}}^{\min} \geq -0.01$. The state-sensitive class allowed kinematic/signed candidates with $\alpha \leq 0.5$, $N_p \geq 2$, $N_c = 0$, $\overline{G}_C \geq 0.10$, $G_C^{\min} \geq -0.10$, $G_\tau^{\min} \geq -0.05$, and $G_{\text{point}}^{\min} \geq -0.02$. If a finite-kernel default candidate had $G_\tau^{\min} < -0.10$, its lower-strength same-family guard required $\alpha \leq 0.75$, $N_p \geq 2$, $N_c = 0$, $\overline{G}_C \geq 0.10$, $G_C^{\min} \geq 0.03$, $G_\tau^{\min} \geq -0.20$, and $G_{\text{point}}^{\min} \geq -0.03$. If a local-short-memory default candidate had $G_\tau^{\min} < -0.20$, its lower-strength same-family guard required $N_p \geq 1$, $N_c = 0$, $\overline{G}_C \geq 0.08$, $G_C^{\min} \geq -0.05$, $G_\tau^{\min} \geq$

-0.30, and $G^{\min}_{\text{point}} \geq -0.02$. The predeclared alanine low-strength probe required kinematic/signed, $\alpha \leq 0.25$, $N_p \geq 2$, $N_c = 0$, $\overline{G}_C \geq 0.10$, $G^{\min}_{C} \geq -0.05$, $G^{\min}_{\tau} \geq -0.20$, and $G^{\min}_{\text{point}} \geq -0.02$; it did not replace the default selector for the reported alanine result. If no override fired, the default selector was retained. Final held-out metrics were never used for selection.

OpenFF surrogate comparison implementation. The four-molecule OpenFF comparison arm predicts the next standardized coordinate from its two most recent values and a Gaussian innovation. It is a second finite-history surrogate implementation, not the additive kinematic/QVA correction used in the primary panels. The two implementations share the PMF-referenced curve-and-relaxation no-harm requirement but not their numerical propagators or fitted parameters. The values are unchanged; the archived implementation determines this model identity.

Table S4: OpenFF/OpenMM stochastic AR(2) finite-history surrogate calculation, reported without numerical alteration and classified according to its archived implementation. Errors are curve NRMSE / relaxation-time relative error; joint gain is the smaller curve and relaxation gain relative to the PMF-only process.

System and coordinate	Scale	PMF-only error	Scalar joint gain	AR(2) generated-trajectory readout
Acetone methyl–methyl	10/4 atoms	1.07 / 581.99%	6.82%	9.89% joint; 0.59% tau error
Toluene methyl–ring centroid	15/7 atoms	1.05 / 606.20%	5.71%	8.24% joint; 1.00% tau error
Ibuprofen carboxyl–isobutyl centroid	33/15 atoms	1.35 / 632.83%	23.73%	42.11% joint; 28.07% tau error
Cholesterol hydroxyl–tail centroid	74/28 atoms	2.14 / 878.83%	39.10%	43.43% joint; 204.50% tau error

Same-data cost comparison.

Over C6, C5, toluene, and acetone, the primary finite-history/surrogate method improved both point-estimate errors in all four comparisons. Its mean joint gain was 10.88%, its best gain was 23.95%, and its minimum gain was 1.93%. The transferred C6-calibrated scalar clock improved both point-estimate errors in three of four comparisons, with corresponding gains of 3.91%, 13.37%, and −11.53%. The former used 21000 pretest fitting/selection rows and 7200 held-out generated/scored correction frames in aggregate: C6 used 6000/1200 rows and frames, and C5, toluene, and acetone each used 5000/2000. The historical benchmark fields 24000 and 7208 record the declared/mixed budget rather than the executed fit and rollout counts. The latter required a 1501-point scalar scan and no corrected rollout. This is a transfer stress test, not a coordinate-specific scalar optimum.

S5. Artifact and verification map

The code archive provides a task-oriented wrapper around the unchanged verification scripts. From the assembled archive root, run `python reproduce/reproduce.py list` to report the evidence level and data parts required for each task. The corresponding `doctor <task>` command checks inputs and dependencies, and `run <task>` invokes the fixed command. The registry distinguishes raw-input replay from table, inventory, and hash verification. The archive further separates three layers that answer different reproducibility questions: source-generation process execution, exact numerical replay from archived inputs, and reconstruction of reported figures and tables. The shortened process task follows all four reported system classes; the full task uses the archived

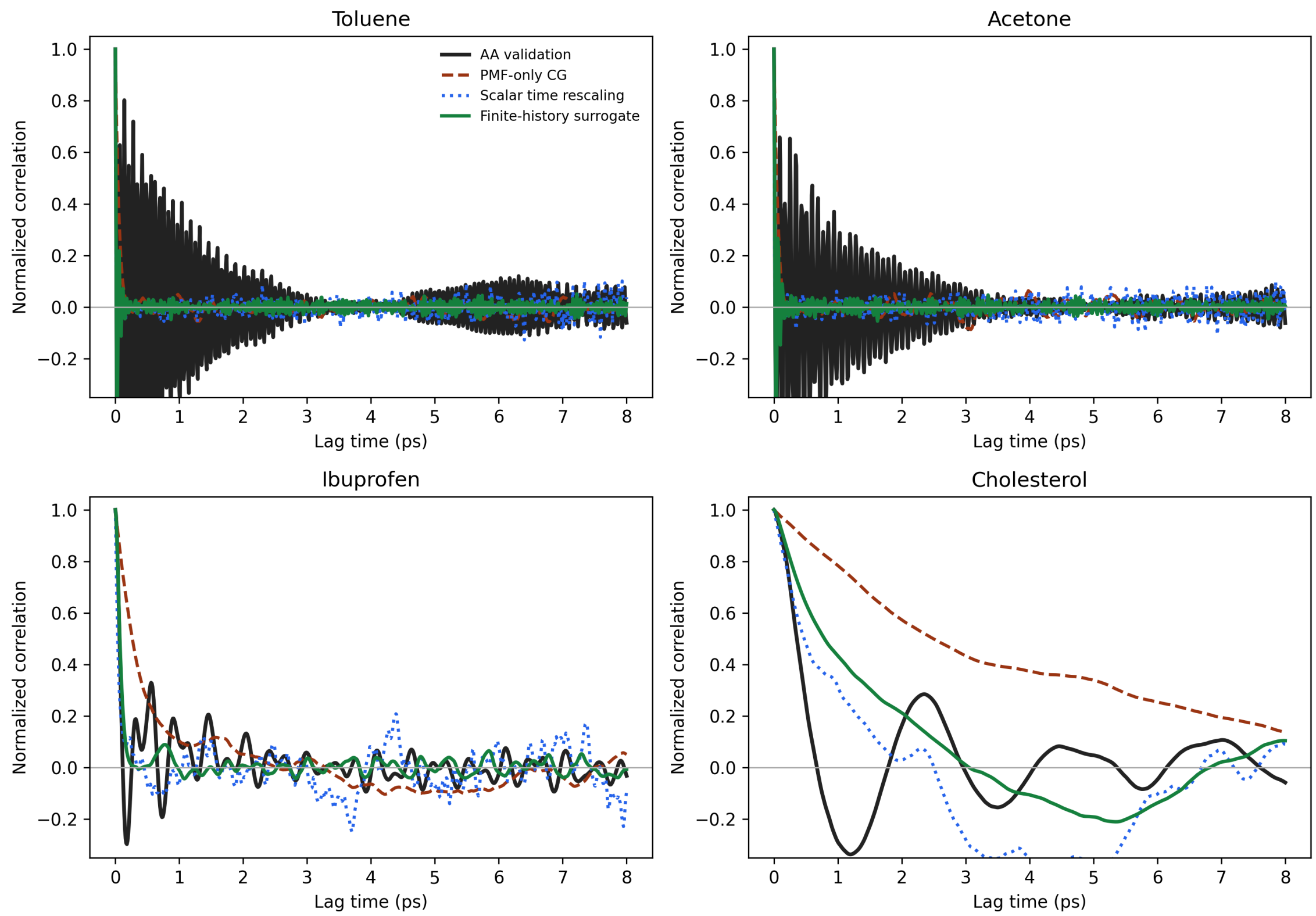

Figure S1: OpenFF/OpenMM finite-history surrogate comparison panel. The AA trajectories and reported generated-coordinate comparisons are unchanged. Inspection of the archived implementation identifies the finite-history-labelled curve in this earlier panel as a separately fitted stochastic AR(2) coordinate process. It is the surrogate arm of the method, not the additive kinematic/QVA update of the primary discrete rollout.

panels, molecular inputs, lengths, strides, and seeds. Cross-platform stochastic trajectories are checked for executable protocol, schema, frame production, and finiteness, while exact manuscript numbers are checked by independent fixed-input tasks. The latter use the versions pinned in `requirements.txt`; the primary replay preflight rejects an incompatible NumPy/SciPy environment before computation.

Table S5: Raw-to-result verification tasks.

Reported evidence	Released input	Verification command	Expected check
Task-oriented entry point	Code/data archive and task registry	`list`; `doctor <task>`; `run <task>`	Required inputs and evidence level are checked before the fixed command runs
Source-generation process	UA alkane panels, generic-molecule driver, alanine inputs, and prepared OpenFF/OpenMM systems	`run experiment-process-smoke`; `run experiment-process-full`	Four source-generation processes execute and produce finite NPZ records with the declared schemas
13-coordinate Figure 3 panel	Thirteen scalar-coordinate NPZ files and the two archived historical drivers	`run legacy-13-figure3-replay`	All 13 dynamic-target values and all 39 seeded correction runs reproduce the 16-column table
Core tables and boundary inventory	Machine-readable result tables, inventories, and hashes	`run finite-history-tables`	Source inventory and all reported values pass
Reported graphics	Released result tables and R/Python figure sources	`run submission-figures`	Main Figures 1–4, the retained SI transfer figure, and the TOC graphic are rebuilt and hashed
C5 output-level check	Released generated-trajectory replay NPZ and manifest	`run c5-raw-replay`	Recomputed trajectory metrics pass; fitting and selection are outside this additional task
C5, acetone, toluene, and alanine finite-history positives	Archived raw coordinate NPZ files, boundary table, memory-fit inventory, and the 39 primary scripts	`run primary-finite-history-replay`	Split sizes, rollout counts, selected family, strength, and three held-out scores pass
Complete PMF-baseline experiments	C6/C5 raw coordinate files, finite-volume PMF transition component, and retained finite-history scripts	`run pmf-same-process-identity`	Paired baseline/corrected replicas reproduce both PMF L1 criteria and both positive dynamical lower bounds
Pre-correction applicability	Acetone C–O and ethanol heavy-atom end-to-end coordinate/force arrays and prespecified thresholds	`run pre-correction-early-stops`	Residual-force metrics, memory-class assignments, $\alpha = 0$, and absence of corrected trajectories pass
OpenFF surrogate comparison panel	Four archived 20000-frame coordinate trajectories and the released analysis script	`run openff-transfer-replay`	PMF-only, scalar, and AR(2) curves, metrics, decisions, and four joint gains pass
Toluene operator bundle	Released raw operator inputs and expected checks	`run toluene-operator-check`	Operator-bundle inputs and reported checks pass

Table S6: Authoritative machine-readable records.

Record	Purpose
`evidence/legacy_13_raw_v1/legacy_13_inventory.json`	Source-archive identity, per-file hashes, arrays, frame counts, and evidence restrictions.
`evidence/legacy_13_raw_v1/legacy_13_uniform_pmf_baseline_results_v2.json`	Per-coordinate static and dynamical metrics from the common detailed-balance analysis.
`evidence/legacy_13_raw_v1/legacy_13_uniform_pmf_baseline_AUDIT_v2.json`	Count, finiteness, static-distribution, and trajectory-hash checks for the 13-coordinate breadth panel.
`evidence/submitted_finite_history/data/core_data/fig3_lower_bound_deployability_data_20260616.csv`	Full 13-coordinate correction decisions, selection strengths, decision reasons, and generated-trajectory gains used in Figure 3.
`evidence/submitted_finite_history_reference_implementation_20260616/`	All 39 Python sources from the primary discrete finite-history implementation, including the baseline-plus-correction rollout, kinematic/QVA/signed-memory fits, and feature-family selectors; `SHA256SUMS_SOURCE.txt` records every released source hash.
`evidence/submitted_finite_history/data/core_data/theory_boundary_validation_20260616.csv`	Authoritative 45-coordinate boundary inventory, including row-level coordinate identity, diagnostic quantities, assigned memory class, and deployment-evidence status.
`evidence/finite_history_primary_replay_v47/`	Runnable subset for the C5, acetone, toluene, and alanine finite-history positives; the verifier checks released-file hashes, split and rollout counts, and the reported selected outcomes from raw coordinate inputs.
`evidence/pmf_same_process_identity_v1/`	Complete C6/C5 PMF-baseline experiments; the verifier refits and regenerates the paired uncorrected/corrected processes and checks the structural and dynamical criteria.
`evidence/pre_correction_early_stop_v1/`	Acetone C–O and ethanol heavy-atom end-to-end applicability calculations; the verifier recomputes residual-memory metrics, assignments, zero strengths, and absence of corrected rollouts.
`evidence/source_generation_reference_20260729/`	Archived process metadata, coordinate panels, molecular inputs, and historical drivers used by the shortened and full source-generation tasks.
`reproduce/rebuild_submission_figures.py`	Single entry point for rebuilding all manuscript, SI, and TOC graphics; it can consume the Figure 3 table produced by the 13-coordinate replay.

All numbers retained in the manuscript tables and figures are generated from complete, verified JSON, CSV, and NPZ artifacts. No value is transcribed from a log file or from an incomplete run.
