# Supplementary material for: No-Harm Missing-Friction Correction for Potential-of-Mean-Force-Matched Coarse Graining
Source: ACS Omega. 2026 Aug 19;11(34):52029–40. doi: 10.1021/acsomega.6c07213 (PMC13625116; doi:10.1021/acsomega.6c07213)
Supplement: Supplementary file 2 [file ao6c07213_si_002.zip › evidence/submitted_finite_history/figures/cgmd/fig1_missing_friction_decision_schematic_20260616_pagefit.pdf]

No-harm missing-friction correction

Recover lost CG friction only when generated trajectories improve.

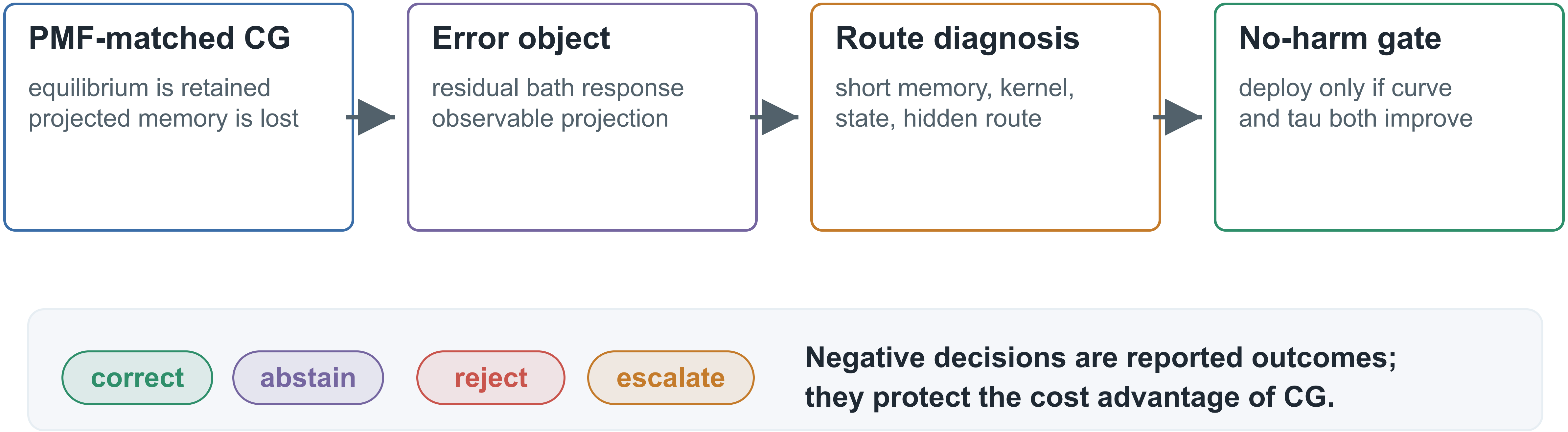

Validation evidence: withheld AA-vs-CG generated trajectories, not residual-force fit alone.
