# Supplementary material for: No-Harm Missing-Friction Correction for Potential-of-Mean-Force-Matched Coarse Graining
Source: ACS Omega. 2026 Aug 19;11(34):52029–40. doi: 10.1021/acsomega.6c07213 (PMC13625116; doi:10.1021/acsomega.6c07213)
Supplement: Supplementary file 2 [file ao6c07213_si_002.zip › evidence/submitted_finite_history/figures/cgmd/fig2_direct_time_validation_20260616_pagefit.pdf]

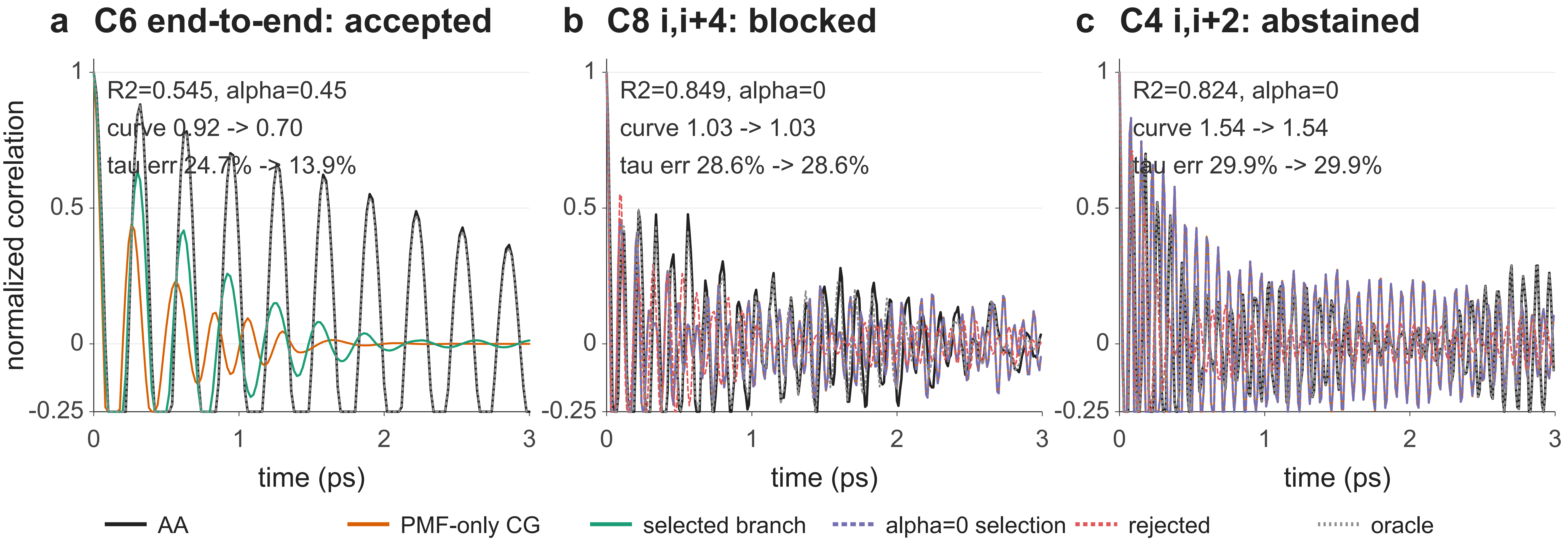

a C6 end-to-end: accepted
b C8 i,i+4: blocked
c C4 i,i+2: abstained
R2=0.545, alpha=0.45
curve 0.92 -> 0.70
tau err 24.7% -> 13.9%
R2=0.849, alpha=0
curve 1.03 -> 1.03
tau err 28.6% -> 28.6%
R2=0.824, alpha=0
curve 1.54 -> 1.54
tau err 29.9% -> 29.9%
normalized correlation
time (ps)
1
0.5
0
-0.25
0
1
2
3
AA
PMF-only CG
selected branch
alpha=0 selection
rejected
oracle
