# Supplementary material for: No-Harm Missing-Friction Correction for Potential-of-Mean-Force-Matched Coarse Graining
Source: ACS Omega. 2026 Aug 19;11(34):52029–40. doi: 10.1021/acsomega.6c07213 (PMC13625116; doi:10.1021/acsomega.6c07213)
Supplement: Supplementary file 2 [file ao6c07213_si_002.zip › evidence/submitted_finite_history/figures/cgmd/fig3_lower_bound_deployability_gate_20260616_pagefit.pdf]

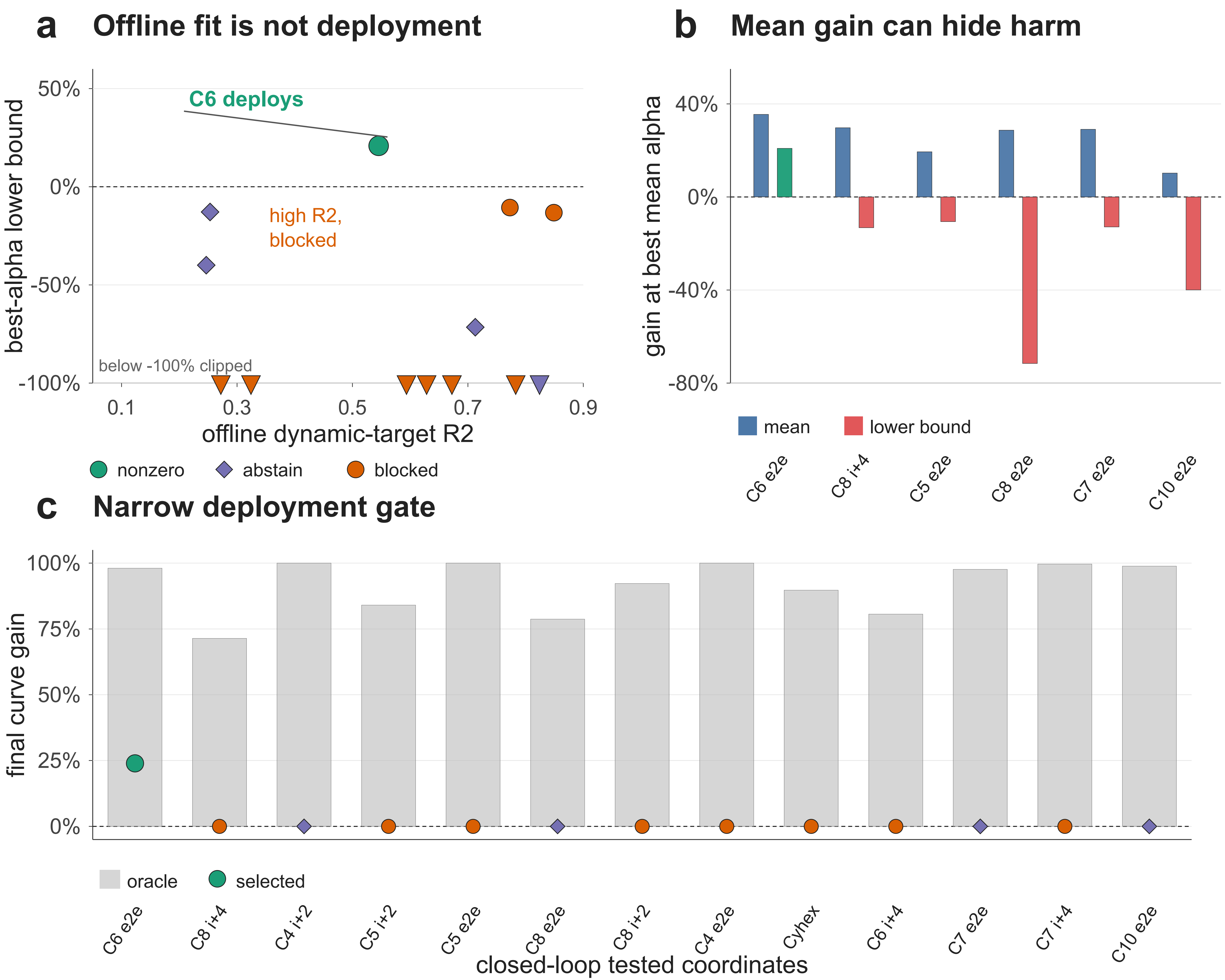

Source: B2_qv_history_L4 late-holdout gate; oracle rows are exact-target upper bounds, not deployable methods.
