# Supplementary material for: No-Harm Missing-Friction Correction for Potential-of-Mean-Force-Matched Coarse Graining
Source: ACS Omega. 2026 Aug 19;11(34):52029–40. doi: 10.1021/acsomega.6c07213 (PMC13625116; doi:10.1021/acsomega.6c07213)
Supplement: Supplementary file 2 [file ao6c07213_si_002.zip › figures/cgmd/fig1_missing_friction_decision_schematic_20260616_pagefit.pdf]

No-harm correction: method and experimental identity

The method is shared; each reported experiment retains its own baseline, data roles, and inference.

(a) Method-to-evidence chain

ABSTRACT METHOD

Define what is corrected

PMF baseline process P_U

finite-history increment alpha x h(H_n)

pre-correction applicability diagnosis

structural and dynamical criteria

→ instantiate

MOLECULAR EXPERIMENTS

Instantiate the method

C6/C5: complete PMF-baseline pairs

four disjoint data roles

acetone/ethanol: applicability cases

alpha = 0 if representation is inadequate

→ evaluate

SCIENTIFIC INFERENCE

Use results in order

1. experiment realizes the method
2. valid experiment tests effectiveness
3. answer the chemical question

apply correction or choose richer model

SAME-PROCESS UPDATE

baseline: Q[n+1] ~ P_U(Q[n], .) corrected: add alpha x h(H_n)

PMF fit, correction fit, validation, and final test remain disjoint.

(b) Experimental identity for repeated coordinate labels

experiment identity = coordinate + baseline/process + data role + candidate/strength + inference rule

PRIMARY EVIDENCE

Complete same-PMF tests

Coordinates: C6 end-to-end; C5 end-to-end

Each test: baseline/corrected share one fitted

PMF transition; data roles remain disjoint

Candidate: independent families; alpha = 0.10

Scoring: 12 validation + 24 final pairs

Inference: PMF tolerance plus two positive

one-sided lower bounds (complete decision)

SUPPORTING EVIDENCE

Breadth and selectivity records

Records: original 13-coordinate panel plus

diagnosis-matched molecular confirmations

Repeated C6/C5 labels denote separate runs

Baselines and strengths remain panel-specific

Submitted outcomes and grids are retained

Inference: breadth/selectivity; qualified or

point-estimate support exactly as reported

POST-DECISION COMPARISON

Cost and relative utility

Records: C6, C5, toluene, and acetone

Comparator: scalar clock 0.855 versus

finite-history or surrogate readout

Inputs: panel-specific reported results

Metrics: G_C / G_tau / joint gain J

Inference: point-estimate cost/utility

comparison, not a new acceptance test

Compare only within a declared experiment record; do not pool effect sizes across the three evidence roles.
