# Supplementary material for: No-Harm Missing-Friction Correction for Potential-of-Mean-Force-Matched Coarse Graining
Source: ACS Omega. 2026 Aug 19;11(34):52029–40. doi: 10.1021/acsomega.6c07213 (PMC13625116; doi:10.1021/acsomega.6c07213)
Supplement: Supplementary file 2 [file ao6c07213_si_002.zip › figures/cgmd/fig3_lower_bound_deployability_print.html]

fig3\_lower\_bound\_deployability\_print


a
Offline fit is not deployment


-100%


-50%


0%


50%

0.1

0.3

0.5

0.7

0.9

C6\_end\_to\_end\_distance | R2=0.5453 | lower=+20.9% | status=nonzero\_deploy

C8\_distance\_i\_i+4 | R2=0.8490 | lower=-13.2% | action=reject

C4\_distance\_i\_i+2 | R2=0.8243 | lower=-1150.2% | status=alpha0\_abstain

C5\_distance\_i\_i+2 | R2=0.7829 | lower=-363.7% | action=reject

C5\_end\_to\_end\_distance | R2=0.7728 | lower=-10.6% | action=reject

C8\_end\_to\_end\_distance | R2=0.7129 | lower=-71.6% | status=alpha0\_abstain

C8\_distance\_i\_i+2 | R2=0.6724 | lower=-806.6% | action=reject

C4\_end\_to\_end\_distance | R2=0.6286 | lower=-252634.0% | action=reject

cyclohexane\_opposite\_carbon\_distance | R2=0.5937 | lower=-5587.6% | action=reject

C6\_distance\_i\_i+4 | R2=0.3242 | lower=-1794.6% | action=reject

C7\_end\_to\_end\_distance | R2=0.2533 | lower=-12.9% | status=alpha0\_abstain

C7\_distance\_i\_i+4 | R2=0.2720 | lower=-477.2% | action=reject

C10\_end\_to\_end\_distance | R2=0.2466 | lower=-39.9% | status=alpha0\_abstain

offline dynamic-target R2
best-alpha lower bound
C6: correct

high R2,
reject
below -100% clipped

correct

abstain

reject
b
Mean gain can hide harm


-80%


-40%


0%


40%


C6 e2e


C8 i+4


C5 e2e


C8 e2e


C7 e2e


C10 e2e
gain at best mean alpha

mean

lower bound
c
Narrow deployment gate


0%


25%


50%


75%


100%


C6 e2e


C8 i+4


C4 i+2


C5 i+2


C5 e2e


C8 e2e


C8 i+2


C4 e2e


Cyhex


C6 i+4


C7 e2e


C7 i+4


C10 e2e
closed-loop tested coordinates
final curve gain

upper bound

correct
Source: fixed late-window validation; upper-bound rows use exact AA targets and are not deployable methods.
